# New Measurements and Calculations on the Kinetics of an Old Reaction: OH + HO_2_ → H_2_O + O_2_

**DOI:** 10.1021/jacsau.3c00110

**Published:** 2023-06-06

**Authors:** Thomas H. Speak, Mark A. Blitz, Diogo J. Medeiros, Paul W. Seakins

**Affiliations:** † School of Chemistry, University of Leeds, Leeds LS2 9JT, U.K.; ‡ National Centre for Atmospheric Science, University of Leeds, Leeds LS2 9JT, U.K.

**Keywords:** kinetics, radical−radical reactions, submerged barriers, tight transition states, water enhancement

## Abstract

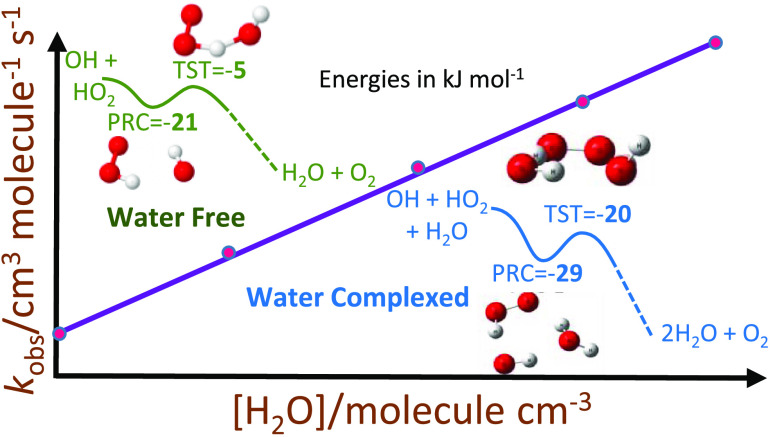

Literature rate coefficients for the prototypical radical–radical reaction  at 298 K vary by close to an order of magnitude; such variations challenge our understanding of fundamental reaction kinetics. We have studied the title reaction at room temperature via the use of laser flash photolysis to generate OH and HO_2_ radicals, monitoring OH by laser-induced fluorescence using two different approaches, looking at the direct reaction and also the perturbation of the slow OH + H_2_O_2_ reaction with radical concentration, and over a wide range of pressures. Both approaches give a consistent measurement of *k*_1,298K_ ∼1 × 10^–11^ cm^3^ molecule^–1^ s^–1^, at the lowest limit of previous determinations. We observe, experimentally, for the first time, a significant enhancement in the rate coefficient in the presence of water, *k*_1,H_2_O, 298K_ = (2.17 ± 0.09) × 10^–28^ cm^6^ molecule^–2^ s^–1^, where the error is statistical at the 1σ level. This result is consistent with previous theoretical calculations, and the effect goes some way to explaining some, but not all, of the variation in previous determinations of *k*_1,298K_. Supporting master equation calculations, using calculated potential energy surfaces at the RCCSD(T)-F12b/CBS//RCCSD/aug-cc-pVTZ and UCCSD(T)/CBS//UCCSD/aug-cc-pVTZ levels, are in agreement with our experimental observations. However, realistic variations in barrier heights and transition state frequencies give a wide range of calculated rate coefficients showing that the current precision and accuracy of calculations are insufficient to resolve the experimental discrepancies. The lower value of *k*_1,298K_ is consistent with experimental observations of the rate coefficient of the related reaction, Cl + HO_2_ → HCl + O_2_. The implications of these results in atmospheric models are discussed.

## Introduction

The reaction of OH with HO_2_ is an important prototypical radical–radical reaction of fundamental interest and, as a chain termination process (i.e., a reaction that removes radicals), is relevant to various aspects of atmospheric^1^ and combustion^2^ chemistry, processes that are driven by free-radical chain reactions often involving OH and HO_2_.
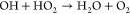
R1It is therefore surprising that, even though radical–radical reactions are difficult to study, literature values of the rate coefficient, *k*_1_, vary from approximately (1–11) × 10^–11^ cm^3^ molecule^–1^ s^–1^; selected examples are shown in Table 1.

**Table 1:** Selection of Previous Experimental Determinations of *k*_1_

authors	10^11^ *k*_1_/cm^3^ molecule^–1^ s^–1^	technique
Assaf and Fittschen^3^	10.2 ± 0.6	laser flash photolysis (LFP), 50 Torr He. (1). Decay of OH(LIF) with excess HO_2_ (CRDS). (2). Variations in OH decays and HO_2_ yields in OH + H_2_O_2_ reaction as initial [OH] varied. Typical *k*_1_′ = 300 s^–1^
Schwab et al.^4^	8 ± 3	discharge flow (DF)/laser magnetic resonance (LMR) for OH and HO_2_ detection, with resonance fluorescence (RF) detection of OH, H, O. 2 Torr He. F + H_2_O and H_2_O_2_, for radical generation. OH with excess HO_2_, typical *k*_1_′ = 20–120 s^–1^
Keyser^5^	11 ± 3	DF/RF at 1 Torr. 254–382 K. NO_2_ added to remove H and O. F + H_2_O/H_2_O_2_ used for radical generation. OH with excess HO_2_, typical *k*_1_′ = 100–300 s^–1^
Sridharan et al.^6^	7.2 ± 0.3	DF/RF at 2 Torr. 252–420 K. OH generation via H + F_2_ = HF + F, F + H_2_O and F + H_2_O_2_ for HO_2_. Can measure atoms via RF and OH via LIF. OH with excess HO_2_, typical *k*_1_′ = 20–120 s^–1^
Sridharan et al.^7^	7.5 ± 1.2	
Wine et al.^8^	∼1	LFP study of OH + H_2_O_2_ monitoring OH via LIF with excess H_2_O_2_, which was directly measured via UV absorption. Dramatically changed initial [OH] and saw no effect on the kinetics, consistent with *k*_1,298K_ ∼1 × 10^–11^ cm^3^ molecule^–1^ s^–1^
Chang and Kaufman^9^	3 ± 2	DF study of OH + O_3_. RF lamp detection of OH and HO_2_ (via NO titration). Looking for deviation in OH traces due to secondary chemistry. OH generated by H + NO_2_

High rate coefficients (*k*_1_ ∼ (7–11) × 10^–11^ cm^3^ molecule^–1^ s^–1^) have been obtained in several discharge flow studies with the generation of both OH and HO_2_ with F-atom-based chemistry, and observation of OH by laser-induced fluorescence (LIF).^4−7^ Most recently, Assaf and Fittschen^3^ used an alternative technique, laser flash photolysis (LFP) of hydrogen peroxide to generate OH and excess HO_2_ (HO_2_ generated indirectly via well characterized Cl/CH_3_OH/O_2_ chemistry), with OH being monitored by LIF and HO_2_ by CRDS (cavity ring down spectroscopy) and determined *k*_1_ = (10.2 ± 0.6) × 10^–11^ cm^3^ molecule^–1^ s^–1^. There have also been several indirect studies involving continuous photolysis,^10^ molecular modulation,^11^ and flash photolysis.^12^ The 2001, IUPAC^13^ recommended value of *k*_1_ = (11 ± 3) × 10^–11^ cm^3^ molecule^–1^ s^–1^ is primarily based on the study of Keyser.^5^

Wine et al.^8^ considered reaction R1 in their study of the reaction of OH with H_2_O_2_
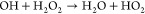
R2They were aware that values of *k*_1_ in the region of 10 × 10^–11^ cm^3^ molecule^–1^ s^–1^ would interfere with their LFP/LIF determinations of *k*_2_. Wine et al. therefore added large additional concentrations of O_3_ to the mix, increasing the initial concentration of both OH and HO_2_ via the reactions of O(^1^D), produced from O_3_ photolysis, with H_2_O_2_
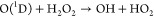
R3Despite increased initial radical concentrations, by a factor of up to 9, they observed no variation in the removal rate of OH, suggesting a considerably lower value for *k*_1_(≤2.2 × 10^–11^ cm^3^ molecule^–1^ s^–1^). A similar lack of dependence of *k*_2_ on radical concentration was reported in a flow tube study by Keyser.^14^ However, Assaf and Fittschen^3^ also studied the reaction R2 using a similar approach to Wine et al. but with much lower [H_2_O_2_] and hence lower pseudo-first-order rate coefficients. In contrast, Assaf and Fittschen did observe significant perturbation of the OH decays and a reduction in the HO_2_ yield consistent with a value of *k*_1_ ∼ 10 × 10^–11^ cm^3^ molecule^–1^ s^–1^.

**Figure 1:**
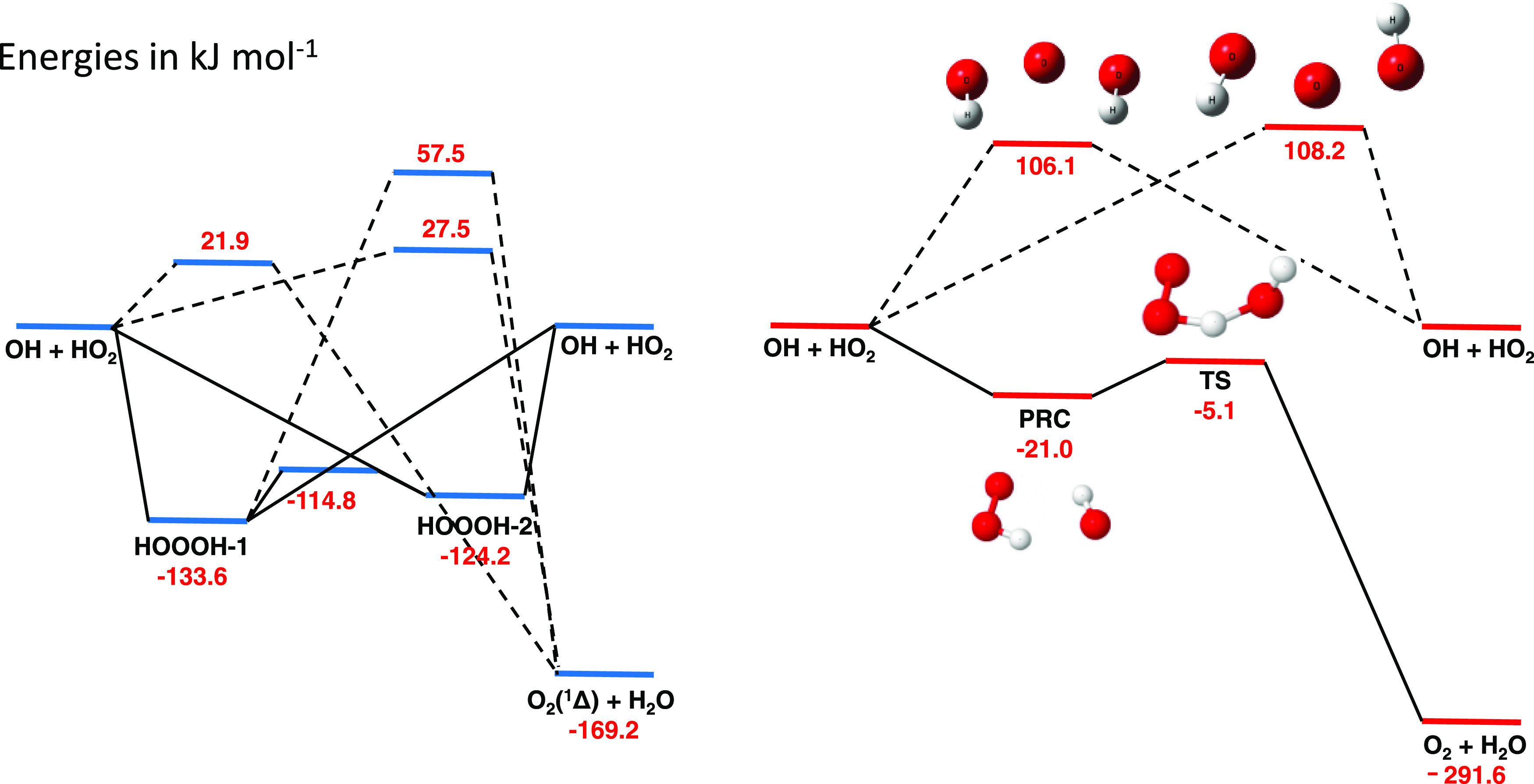
Singlet (left) and triplet (right) surfaces for the uncatalyzed reaction of OH and HO_2_. The energies, in kJ mol^–1^, for the singlet surface were calculated at the CCSD(T)/CBS level with the anharmonic zero point energy (ZPE) corrections calculated using the same method as used to calculate the structures (M06-2X and MP2 with the 6-311++g(3df,3pd) basis set). Triplet energy levels were calculated at the RCCSD(T)-F12b/CBS//RCCSD/aug-cc-pVTZ level.

Due to both significant practical and fundamental interest, there have been several theoretical studies of reaction R1.^15−23^ All studies agree that the reaction predominantly takes place on the triplet potential energy surface (PES), as shown in Figure 1, via the formation of a pre-reaction complex (PRC), stabilized ca. 13–24 kJ mol^–1^ below the entrance channel, with a submerged, but relatively tight transition state (−7 to −12 kJ mol^–1^ below the entrance channel) leading to products. Although the primary focus of several studies was kinetics at high temperatures, virtually all theoretical studies report room-temperature rate coefficients for *k*_1_ of ca. 1–7 × 10^–11^ cm^3^ molecule^–1^ s^–1^. Several recent studies predict that *k*_1_ has a significant water dependence, although none has been reported from experimental measurements despite significant variations in [H_2_O].^15,18^

Although reaction via the triplet state is dominant, the excited singlet state can play a role. Badenes et al.^24^ calculated a high barrier (56.8 kJ mol^–1^) from the HOOOH complex to the abstraction projects and hence only considered recombination. Recombination only plays a significant role at very high pressures. More recently, Lu et al.^25^ found a pathway on the singlet surface with a slightly submerged barrier (ca. −4 kJ mol^–1^) and suggested that reaction on the singlet surface could contribute to ∼10% of the reaction.

Our initial interest in reaction R1 was sparked by comments on our paper outlining the development of a new LIF instrument for OH and HO_2_ detection.^26^ We used both reaction R2 and reaction R4 to internally calibrate our OH and HO_2_ LIF signals to allow us to determine HO_2_ yields from OH-initiated reactions.
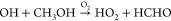
R4Both methods gave consistent results, but the slower reaction R2 calibration reaction should show a dependence on radical concentration if *k*_1_ ∼10 × 10^–11^ cm^3^ molecule^–1^ s^–1^.

In this paper, we present three laser flash photolysis/laser-induced fluorescence experiments designed to determine *k*_1_ over a range of conditions:

(1). Studying the variation of *k*_2_ via LIF observation of OH using a conventional slow flow LFP/LIF system at pressures of 75–200 Torr.^27,28^
(2). Studying the variation of *k*_2_ via LIF observation of both OH and HO_2_ at high pressures (∼1800 Torr N_2_) as a function of initial radical concentration using our new instrumentation.^26^
(3). Photolyzing water in the presence of O_2_ to generate equal concentrations of OH and HO_2_ at high pressures (∼1800 Torr N_2_) and with varying concentrations of water to investigate the predicted water dependence of *k*_1_.

We support our experimental observations with calculations of the PES for R1 and analogous reactions and calculate rate coefficients using the master equation code MESMER.^29^

## Methods

### Experimental Section

**Figure 2:**
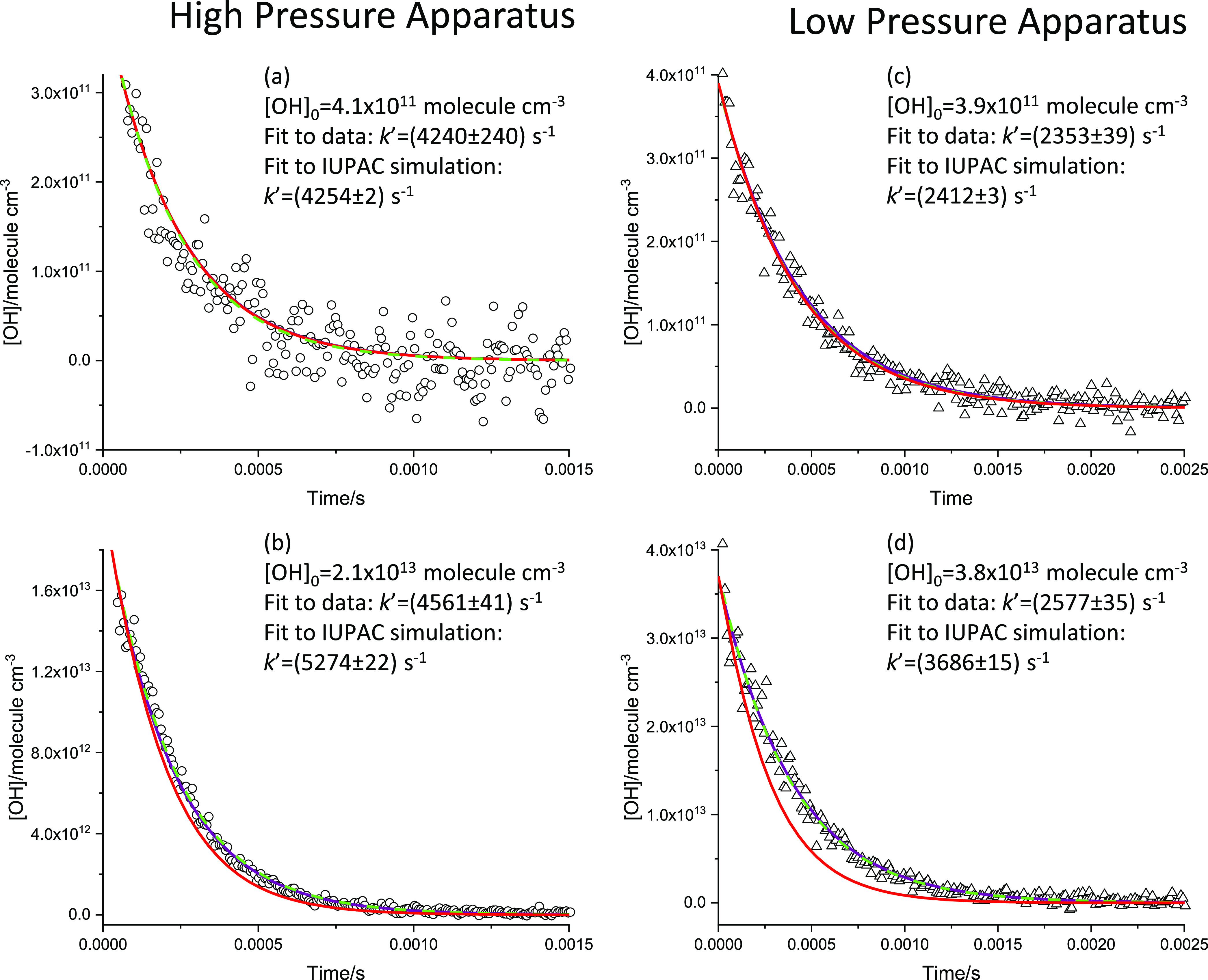
Four example traces with varying laser power in the two different experimental setups. Traces (a, b) from the high-pressure apparatus (1800 Torr of N_2_ at 298 K, [H_2_O_2_] = 2.5 × 10^15^ molecule cm^–3^). Traces (c, d) from the conventional low-pressure apparatus (200 Torr of Ar at 298 K, [H_2_O_2_] = 1.4 × 10^15^ molecule cm^–3^). These traces differ in their initial OH concentration ranging from (a) [OH]_0_ = 4.1 × 10^11^ molecule cm^–3^ to (d) [OH]_0_ = 3.8 × 10^13^ molecule cm^–3^. ○, Data points from high-pressure apparatus; (Δ) data points from low-pressure apparatus; (blue line), fit from global analysis; (green line), exponential fit to that single data trace; (red line) simulation using [OH]_0_ and IUPAC rate coefficients.

All studies were carried out in two slow-flow, laser flash photolysis/laser-induced fluorescence systems (LFP/LIF), one at high pressures (typically ∼1800 Torr N_2_) and the other at lower pressures of 75–200 Torr (Ar or N_2_). Hydrogen peroxide (50% v/v with H_2_O, Sigma-Aldrich) photolysis at 248 nm was used as the photolysis source of OH radicals for most experiments. This process is well characterized and produces only ground vibrational state OH.
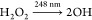
P1Calibrated energy meters were used to measure the laser fluence and hence, in combination with known precursor concentrations, estimate the initial radical concentrations. LIF was used to detect OH in both systems and details are given below. The time delay between the photolysis pulse and the LIF probe pulse was systematically varied to build up OH temporal profiles. Examples of typical OH decay traces can be seen in Figure 2. For both systems, hydrogen peroxide from a thermostatted bubbler was entrained into a flow of argon and then into the reaction chamber. Pressures in the reaction chamber were controlled by throttling the exit valve and were measured using the appropriate capacitance manometer.

### High-Pressure System

Details on the high-pressure system can be found in Stone et al.^30^ and Speak et al.^26^ and a schematic of the apparatus is shown in Figure S1. The pre-mixed gases (H_2_O_2_, H_2_O, and N_2_) were flowed through a high-pressure tube. Photolysis (Lambda Physik Compex 200, output fluence 0.5–120 mJ cm^–2^) occurred along the axis of the high-pressure flow tube and photolysis repetition rates were varied to ensure that there were no effects on the kinetics from multiple photolyses. At the end of the tube, a portion of the gas mixture was sampled through a 1 mm diameter pinhole into a low-pressure (∼3 Torr) region forming a jet. In this region, OH was monitored via LIF, exciting the radicals at 282 nm (Sirah dye laser operating on Rhodamine 6G, pumped by 532 nm output of a Quantel Q Smart 850 Nd:YAG laser) to *v* = 1 in the upper electronic state and monitoring the resultant fluorescence at 308 nm through a narrow band filter (308 ± 5 nm, Barr Associates) coupled with a photomultiplier tube (PMT, PerkinElmer C1943P), mounted perpendicular to the probe laser and the jet. At the point at which the jet breaks down, NO was injected to convert HO_2_ into OH
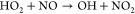
R5with the OH produced monitored in a second LIF cell with duplicate fluorescence collecting apparatus. Flows of NO were alternated with N_2_ to allow the subtraction of the residual OH reaching the second LIF cell.

### Low-Pressure System

The low-pressure system was a conventional slow flow system and details can be found in Onel et al.^31^ and Glowacki et al.^28^ and an apparatus schematic is shown in Figure S2. Argon (200 Torr) was predominantly used as the bath gas, but some experiments were carried out with nitrogen (75 Torr) as the bath gas. The photolysis laser (Lambda Physik LPX 200), probe laser (Continuum Precision II Nd:YAG pumping a Sirah dye laser operating with DCM dye), and fluorescence detection axes were mutually perpendicular. For this system, OH was probed via excitation at 308 nm to *v* = 0 in the first electronically excited state and the PMT (PerkinElmer C1943P) detected the resonant fluorescence.

Typically, experiments were run at 10 Hz, but checks were carried out at 2 Hz to ensure that multiple photolyses were not an issue (see Figure S4). Wire gauze filters were used to attenuate the photolysis energy from ∼100 mJ pulse^–1^ to ∼1 mJ pulse^–1^; with identical OH precursor concentrations, this resulted in a decrease of approximately a factor 100 in the initial [OH].

### Supporting Calculations

Supporting rate theory and *ab initio* calculations were undertaken to complement the experimental studies. *Ab initio* calculations for the PES for reaction R1 were performed at various levels of theory using Gaussian 09^32^ and MOLPRO 2012^33^ in order to generate energies and vibrational frequencies required for the master equation rate theory calculations. Further details on the *ab initio* calculations can be found in the SI, Section S2.

Rate coefficients are calculated using a master equation analysis of the pre-reaction complex (PRC). The capture rate coefficient for entry into the PRC is estimated using the methods of West et al.^34^ The micro-canonical rate coefficients for re-dissociation of the complex back to reagents is determined by an inverse Laplace transform (ILT) method. The rate of the forward reaction over the well-defined transition state to products is determined by a conventional RRKM analysis. The RRKM microcanonical formulation for a unimolecular reaction is
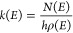
E1where *N*(*E*) is the number of accessible states in the TS and ρ(*E*) is the density of states of the pre-reaction complex. The calculations allow for the optical isomers of the transition state as discussed by Monge-Palacios and Sarathy.^17^ Calculations were implemented by the MESMER software package.^29^ An example MESMER input file can be found in the SI, Section S9.

## Results

Figure 2a,b shows examples of typical OH traces following the photolysis of H_2_O_2_ at 248 nm to generate OH radicals and their subsequent removal by reaction R2 in the high-pressure system. The traces represent a wide range of initial [OH] (overall concentrations varied from 1.1 × 10^11^ to 2.6 × 10^13^ molecule cm^–3^) as evidenced by the varying signal to noise. Over 54 OH and 12 HO_2_ traces were taken and fitted globally to return a value of *k*_1_ = (1.34 ± 0.14) × 10^–11^ cm^3^ molecule^–1^ s^–1^, where the error is statistical at the 1σ level. In Figure 2, we show the results of the global analysis (purple line), along with individual exponential fits of each trace (green line, for most traces the global and local fits overlap) and the simulated profiles for *k*_1_ = 11 × 10^–11^ cm^3^ molecule^–1^ s^–1^^13^ (red line). In agreement with Wine et al., there is very little dependence of the pseudo-first-order rate coefficient, *k*_1_′, with initial [OH] (varied from 4.1 × 10^11^ to 2.1 × 10^13^ molecule cm^–3^ in the figure plots) and certainly much less than predicted based on the IUPAC recommended value (red lines).

Section S3 in the SI also contains additional example traces from similar studies on reaction R2 carried out in both apparatuses over a range of [OH]_0_ and with varying laser repetition rates. Example HO_2_ traces and additional information on monitoring HO_2_ can be found in Figure S5 in the SI (Section S4). In the low-pressure studies, a set of wire meshes was used to vary the photolysis laser fluence from 120 to 1 mJ cm^–2^ pulse^–1^. A series of back-to-back low- and high-laser-power experiments were made; analysis of the 47 resulting traces (32 at 200 Torr of argon and 15 at 75 Torr of N_2_) gave *k*_1_ = (1.00 ± 0.32) × 10^–11^ cm^3^ molecule^–1^ s^–1^. Examples of experimental traces with low and high initial [OH] are shown in Figure 2c,d.

**Figure 3:**
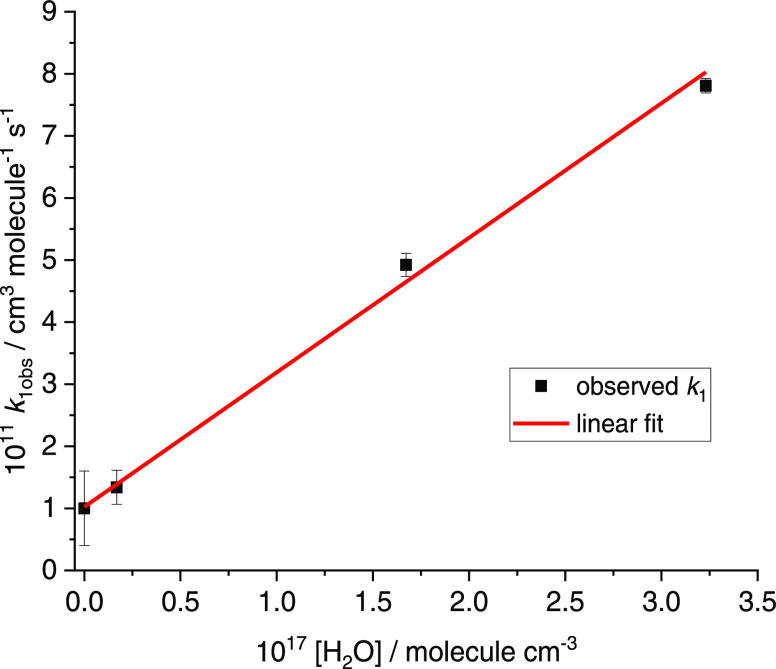
Plot of the observed bimolecular rate coefficient for OH and HO_2_ (sum of both catalyzed and uncatalyzed) against water concentration. The black points (error bars 2σ) are the experimental values and the red line is a linear fit giving *k*_1,H_2_O_ = (2.17 ± 0.09) × 10^–28^ cm^6^ molecule^–2^ s^–1^.

For the high-pressure water photolysis experiments in the presence of oxygen, equal initial concentrations of OH and HO_2_ are obtained via
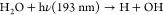
P2
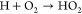
R6Significantly higher values of *k*_1_ were observed, but crucially, the observed rate coefficients varied with [H_2_O] as shown in Figure 3. Extrapolating the observed rate coefficient to zero water produces a value of *k*_1_ = (1.02 ± 0.11) × 10^–11^ cm^3^ molecule^–1^ s^–1^, in good agreement with our previously discussed results on the variation of the kinetics of the OH + H_2_O_2_ reaction as a function of initial OH concentration. Note that in the studies based on R2, some water would have been present in the system, but only at concentrations similar to that of the H_2_O_2_, 1–5 × 10^15^ molecule cm^–3^. Therefore, the expected enhancement based on the presence of water is only ∼1 × 10^–12^ cm^3^ molecule^–1^ s^–1^, i.e., less than the uncertainty in those measurements.

The values of *k*_1_ depend sensitively on the initial radical concentration generated. Initial radical concentrations were calculated from measured laser fluences, precursor concentrations, and literature values for the absorption cross sections and quantum yields.^13^ It is recognized that there are errors associated with these values, especially in accurately measuring low laser powers and in determining precursor concentrations. Concentrations of H_2_O_2_ could be constrained by the established value of *k*_2_ ((1.7 ± 0.4) × 10^–12^ cm^3^ molecule^–1^ s^–1^)^13^ at low radical concentrations, water concentrations were directly measured with a dew point hygrometer. We estimate that radical concentrations could have an uncertainty of ∼25%, but we would need to be out by a factor of  in the initial radical concentration of OH and HO_2_ to bring our water-free results into agreement with the IUPAC recommendation.

Figure 1 shows our potential energy surface (PES) for the singlet and triplet surfaces of reaction R1. For the singlet reaction channel, calculations at the CCSD(T)/CBS//MP2/6-311++g(3df,3pd) and CCSD(T)/CBS//M06-2X/6-311++g(3df,3pd) levels were in good agreement. However, for the triplet reaction surface, it was found that the abstraction transition state structure was less certain and highly method-dependent. Table 2 shows the variation in the key parameters required for the rate coefficient calculations (a full list of frequencies can be found in the SI, Section S2 and Table S1) and the resulting room temperature rate coefficients. These different structures yielded not just differing activation barriers but also different low-frequency vibrational modes. Further work to evaluate this variability was carried out by repeating the UMP2 and M062X structures with the aug-cc-pVTZ basis sets along with calculating UCCSD/ aug-cc-pVTZ structures in Gaussian 09 and RMP2/aug-cc-pVTZ and RCCSD-F12a/aug-cc-pVTZ structures in Molpro 2012. The RMP2 and RCCSD-F12a calculations in Molpro were carried out to evaluate the influence of spin contamination as an explanation for the differing structures that had been calculated. All of these structures were then combined with extrapolated CCSD(T) single-point energies.

**Table 2:** Key Information for the Calculation of Rate Coefficients for the OH + HO_2_ Water Free Triplet Abstraction Surface Calculated at Various Levels of Theory^a^

method	A	B	C	D	E	F	G
*k*_bim,300K_ cm^3^ molecule^–1^ s^–1^	1.1 × 10^–11^	4.7 × 10^–11^	1.2 × 10^–11^	9.2 × 10^–11^	4.4 × 10^–12^	5.8 × 10^–12^	2.4 × 10^–12^
TS, imaginary cm^–1^	2658	2020	1387	1524	2441	2521	3162
TS ν_1_ cm^–1^^b^	251	110	205	101	397	395	292
TS ν_2_ cm^–1^	520	195	388	164	503	493	566
TS ν_3_ cm^–1^	742	458	551	475	693	694	736
TS ν_4_ cm^–1^	775	656	857	640	770	773	765
E TS kJ mol^–1^	–5.12	–6.11	–6.71	–10.97	–2.06	–3.31	–2.98
PRC ν_1_ cm^–1^	147	148	195	206	151	149	178
PRC ν_2_ cm^–1^	204	205	217	233	225	213	227
PRC ν_3_ cm^–1^	250	257	238	252	307	286	326
PRC ν_4_ cm^–1^	422	451	413	350	503	491	499
E PRC kJ mol^–1^	–21.00	–20.78	–21.65	–21.58	–20.51	–20.69	–19.64

a A = RCCSD(T)-F12b/CBS//RCCSD/aug-cc-pVTZ, B = UCCSD(T)/CBS//UCCSD/aug-cc-pVTZ,C = UCCSD(T)/CBS//UM06-2X/aug-cc-pVTZ, D = UCCSD(T)/CBS//UM06-2X/6-311++g(3df,3pd), E = UCCSD(T)/CBS//UMP2/aug-cc-pVTZ, F = UCCSD(T)/CBS//UMP2/6-311++g(3df,3pd), G = RCCSD(T)-F12b/CBS//RMP2/aug-cc-pVTZ.

b Only the lowest frequencies are significant for rate coefficient calculations. A full listing of frequencies can be found in the SI, Table S1.

We have also carried out a range of calculations to investigate the observed water dependence in this study and predicted in earlier theoretical studies. As a first step, we calculated the PES for H_2_O–HO_2_ complexation at the CCSD level (Section S5 and Figure S7), then, using MESMER we were able to calculate the equilibrium constant for complexation. As shown in Table S3, our calculated equilibrium constants are in good agreement with the experimental determination of Kanno et al.^35^ From the measured water concentration, we are now able to calculate the [HO_2_–H_2_O] and an equivalent version of Figure 3, now showing *k*_1_′ vs [HO_2_–H_2_O] is shown in Figure S8.

**Figure 4:**
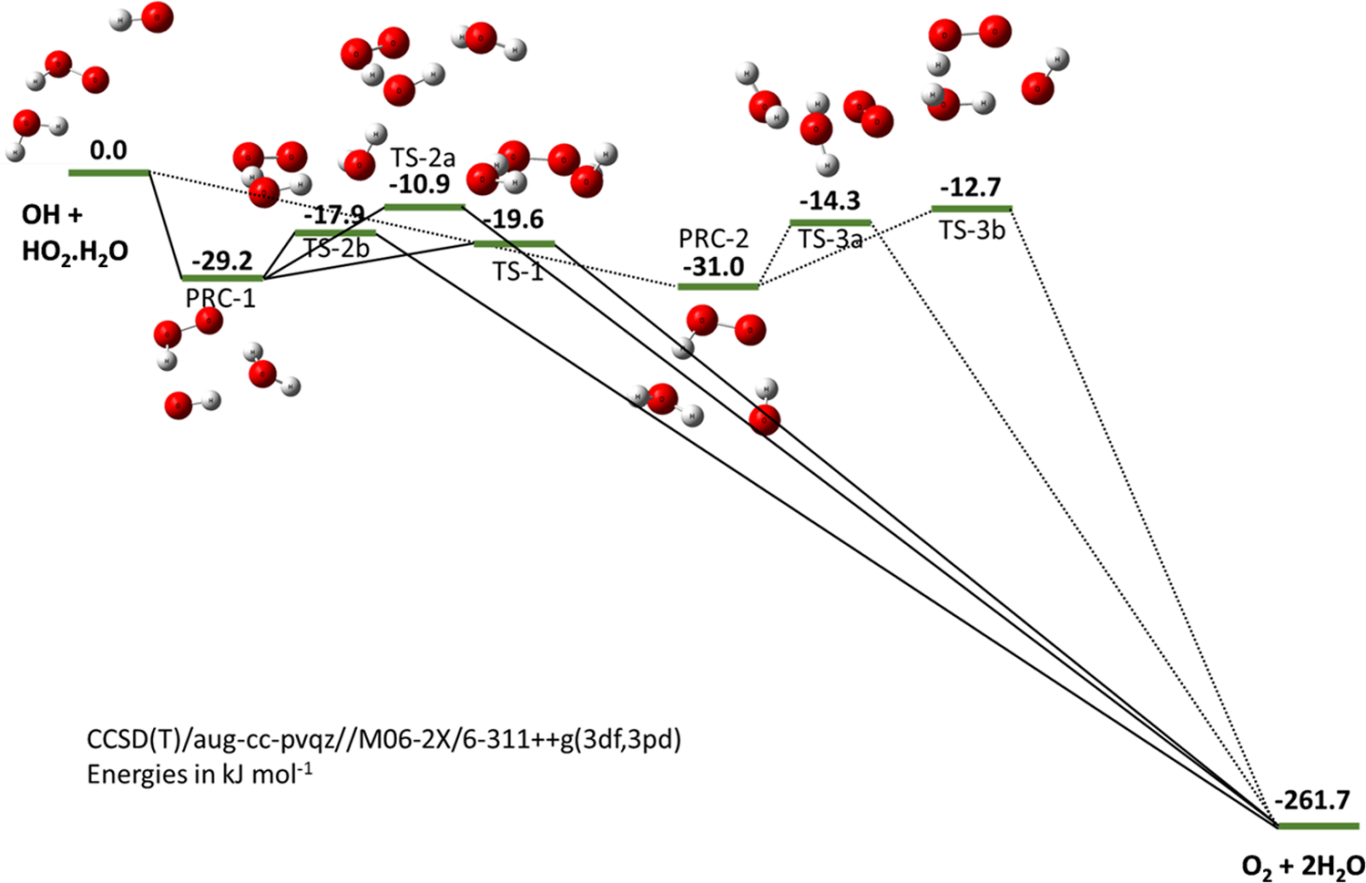
PES for the water-complexed system. Calculations were performed at the CCSD(T)/aug-cc-pvqz//M06-2X/6-311++g(3df,3pd) level. Note that the wells and transition states are significantly lower than for the uncomplexed system (Figure 1).

The PES for the water-catalyzed surface is shown in Figure 4, where values are taken relative to the reactants OH and HO_2_ complexed to water. In contrast to the uncatalyzed PES (Figure 1), there are two PRC, both of which are deeper (−29.2 or −31.0 vs −21.0 kJ mol^–1^ (Method A)). There are multiple pathways from either PRC to the products, and for each PRC, the lowest submerged barrier is significantly lower than for the un-complexed reaction (−19.6 or −14.3 vs −5.1 kJ mol^–1^ (Method A)) accounting for the significant increase in rate coefficient for the reaction of OH with water-complexed HO_2_.

## Discussion

### Experimental Measurements

Even accounting for uncertainties associated with the initial radical concentration in these studies, there are large differences in the water-free rate coefficient for reaction R1 between this study and many other radical–radical studies. Our results are in good agreement with earlier studies on the OH + H_2_O_2_reaction R2^8,14^ where there appears to be no interference on R2 by reaction R1, setting upper limits of *k*_1_ ≤ 1–2 × 10^–11^ cm^3^ molecule^–1^ s^–1^. Our water photolysis studies confirm the predictions of Zhang et al. that complexation of HO_2_ and water significantly enhances the rate coefficient for reaction R1.

Radical–radical reactions are difficult to study, and the wide range of values for *k*_1_ reported over the last 50 years suggest that many studies are subject to various systematic errors. Previous studies of reaction R1 appear to have been carefully carried out, with consideration of secondary chemistry. We cannot provide an explanation for all of the variations in *k*_1_, and, although we have used a range of approaches in our study, we cannot rule out unknown systematic errors in our own work.

While the interference of tropospheric levels of water has a significant impact on the observed value of *k*_1_, water dependence cannot explain the difference with previous low-pressure flow tube measurements. According to Figure 3, [H_2_O] needs to be ∼3.5 × 10^17^ molecule cm^–3^ in order to give *k*_1_ ∼ 10 × 10^–11^ cm^3^ molecule^–1^ s^–1^ which is several orders of magnitude higher than typical values in a low-pressure flow tube even when F + H_2_O is used to generate OH reactant radicals. However, the presence of water can partially explain the high values measured in the higher-pressure photolysis studies of Braun et al.,^12^ De More^10^ and Cox et al.^11^ (further discussion can be found in the SI, Section S6).

The IUPAC recommendation is primarily based on the flow tube study of Keyser.^5^ This value, *k*_1_ = (11 ± 3) × 10^–11^ cm^3^ molecule^–1^ s^–1^, is almost double that from a similar earlier study by Keyser^36^ and a study by Sridharan et al.^6^ and higher than another, higher-pressure, flow tube study by Schwab et al.^4^ (Table 1). Keyser attributed the new higher value due to the presence of atomic species, O and H, which can regenerate OH via secondary chemistry^13^
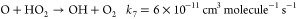
R7
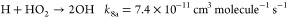
R8a

However, both the flow tube systems of Schwab et al.^4^ and Sridharan et al.^6^ were specifically set up to monitor atomic species as well as OH, so it would be surprising if there were significant atomic impurities in these studies. Additionally, although both the Keyser and Sridharan et al. studies report negative activation energies, the activation energy of the Keyser study is almost a factor of 2 lower, and both temperature dependencies are significantly lower than theoretical predictions. Could heterogeneous chemistry be responsible for the variation in rate coefficients between such studies and with this study? The flow tube studies do use water for radical generation and while the water concentration is insufficient to enhance the gas phase chemistry, water could promote a heterogeneous process.

Heterogeneous chemistry cannot explain the laser flash photolysis results of Assaf and Fittschen.^3^ Their study involves two approaches, a direct generation of OH and HO_2_ following photolysis of H_2_O_2_/(COCl)_2_/CH_3_OH/O_2_ mixtures to generate OH directly and HO_2_ by the well understood Cl/CH_3_OH/O_2_ system giving an excess of HO_2_; OH decays were followed by LIF and the concentration of HO_2_ was monitored by IR cavity ring-down spectroscopy (CRDS). Second, by probing the secondary chemistry in the OH + H_2_O_2_ reaction which is discussed below. We have no explanation for the difference between our values and the direct study of reaction R1 by Assaf and Fittschen, in both cases HO_2_ is monitored and the second-order decay of HO_2_ constrains the [HO_2_].

There are a range of indirect determinations of *k*_1_ via a study of reaction R2, the reaction of OH with H_2_O_2_. At high conversions of OH to HO_2_, reaction R1 can start to compete with reaction R2 for OH radicals, enhancing the loss of OH. As reaction R2 is relatively slow (*k*_2,298K_ = 1.7 × 10^–12^ cm^3^ molecule^–1^ s^–1^)^13^ and was the subject of some uncertainty in the early 1980s, studies of reaction R2 took great care in either keeping radical concentrations very low, or checking that values of *k*_2_ were independent of the initial radical concentration.

One of the earliest such studies was a flow tube study by Keyser.^14^ The value of *k*_2_ reported in this work (*k*_2,298K_ = (1.64 ± 0.32) × 10^–12^ cm^3^ molecule^–1^ s^–1^) is in excellent agreement with other studies. Typical pseudo-first-order decays were 100–200 s^–1^ with a statistical error in the linearized slopes of 1–2% and the reaction was followed over several lifetimes, therefore for a substantial part of the OH decay, [HO_2_] > [OH]. At the highest radical concentrations used (6.6 × 10^11^ molecule cm^–3^) R1 should have contributed significantly to the OH decay (∼25% enhancement) for *k*_1_ ∼ 10 × 10^–11^ cm^3^ molecule^–1^ s^–1^, but no effect was observed.

Reaction R2 was also studied in flash photolysis experiments. Kurylo et al.^37^ varied initial radical concentrations by a factor of 5 and reported no variation in the pseudo-first-order rate coefficient. However, their initial OH radical concentration was low so that even with *k*_1_ ∼ 10 × 10^–11^ cm^3^ molecule^–1^ s^–1^, the effect on *k*_2_′ would be close to the detection limit.

As discussed in the introduction, Wine et al.^8^ took a more robust approach, generating large initial concentrations of OH and HO_2_ via ozone photolysis to produce O(^1^D) followed by reaction R3
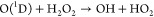
R3No variation in *k*_2_′ was observed. We have simulated the data in the Wine et al. study and this independence of the observed *k*_2_′ is again consistent with *k*_1_ = 1 × 10^–11^ cm^3^ molecule^–1^ s^–1^.

In contrast, Assaf and Fittschen^3^ did see a significant variation in *k*_2_′ in their study, in line with *k*_1_ = 10 × 10^–11^ cm^3^ molecule^–1^ s^–1^. Once again, careful checks have been carried out and the results seem to be consistent across a range of conditions, so there is no obvious explanation for the difference in *k*_1_. The concentration of H_2_O_2_ used was significantly lower than in this and other LFP studies so that *k*_2_′ is around 300 s^–1^, raising the possibility that other secondary reactions could contribute. The effect of reaction R1 should have an induction period while [HO_2_] builds up, but this is not discernible in the traces shown.

In summary, our experimental determination of *k*_1_ contradicts a large number of direct and indirect studies of the OH + HO_2_ reaction (comparisons with other studies are covered in more detail in Section S6 in the SI). We can partially rationalize some differences due to water effects and differences between the flow tube studies suggest that systematic errors (possibly due to heterogeneous processes) may be present. Our values for *k*_1_ are in good agreement with at least two experimental studies of the OH + H_2_O_2_ reaction,^8,14^ where significant variations of *k*_2_′ should have been observed if *k*_1_ is ∼10 times our observed value, but were not. Although such studies are a less direct way of determining *k*_1_, experimentally they are much simpler to carry out, and so less subject to systematic errors. In the absence of any definitive experimental evidence, we now turn to calculations and see what light they cast on Reaction R1.

### Theoretical Calculations

Table 3 summarizes previous calculations on Reaction R1. Qualitatively, all recent calculations produce a very similar PES characterized by the formation of a weakly bound pre-reaction complex (PRC) followed by the crossing of a relatively “tight” transition state to form products. The variation in key PES parameters illustrated in Table 3 was the driving force in our systematic exploration of reaction R1 at various levels of theory. As with the experimental values, our calculations of *k*_1_ are lower than those in the literature; however, virtually all of the literature values are at least approximately a factor 2–5 lower than the IUPAC recommended value.

**Table 3:** PES Parameters for Reaction R1 from Various Studies

references	theory level	energy of pre-reaction complex (kJ mol^–1^)	energy of transition state (kJ mol^–1^)	10^11^ *k*_1_/cm^3^ molecule^–1^ s^–1^
Liu et al.^22^	CCSD(T)-F12a/AVTZ	–24.5	–6.1	16.6 RPMD^a^
				∼3.5 QD^b^
Liu et al.^19^	CCSD(T)-F12a/AVTZ	–13.6	–6.7	∼3.5
Zhang et al.^18^	CCSD(T) aug-cc pVTZ/	–20.9	–12.5	6.64
Monge-Palacios and Sarathy^17^	CCSD(T)W3X	–13.4	–10.0	5.6
Burke et al.^16^	MS-CASPT2/CBS//MS-CASPT2/aug-cc-pVTZ			5
Zhang et al.^15^	CCSD(T) aug-cc pVTZ	–24.0	–2.8	2.3
this work—method A	RCCSD(T)-F12b/CBS// RCCSD/aug-cc-pVTZ	–21.0	–5.1	1.1
this work—method B	UCCSD(T)/CBS//UCCSD/aug-cc-pVTZ	–20.8	–6.1	4.7

a Calculated using ring polymer molecular dynamics.

b Calculated using quantum dynamics.

Once the PRC is formed, there is a competition between re-dissociation and product formation. Although the barrier to product formation is lower, this transition state is tighter compared to re-dissociation. The RRKM microcanonical formulation for a unimolecular reaction is
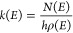
E1awhere the terms were defined earlier. The number of states, *N*(*E*), in the loose TS for re-dissociation dominates over the number of available states in the tighter TS for product formation and therefore only a fraction of the PRC complexes formed lead to reaction.

Our calculated value of *k*_1_ depends on a number of factors: the capture rate coefficient for PRC formation (relatively unimportant at temperatures of 300 K and above), the depth of the PRC and the TS to products, and the vibrational frequencies (especially low-frequency vibrations and hindered rotors) of the PRC and TS. In Table 2 we summarized the values of *k*_1_ as a function of *ab initio* methodology. While there is some variation in *k*_1_, unrealistic parameter values are required to generate values of *k*_1_ greater than 5 × 10^–11^ cm^3^ molecule^–1^ s^–1^. The value of *k*_1_ calculated *via* MESMER can be considered as a lower limit in that it assumes a fully statistical distribution of energy. Trajectory calculations, such as those run by Liu et al.,^19,22^ have no such limitations, although they do require a complete PES. Liu et al.^19^ unfortunately do not compare their trajectory calculations with transition state theory on the same PES. The more recent study of Liu et al.^22^ is based on their earlier PES, but with a deeper pre-reaction complex. The rate coefficient calculated using ring polymer molecular dynamics (RPMD) produces a value for *k*_1,300K_ of 1.66 × 10^–10^ cm^3^ molecule^–1^ s^–1^, in agreement with the recommended IUPAC value. However, Liu et al. note that their RPMD calculated rate coefficient is approximately a factor 3 greater than that calculated by Song et al.^23^ using the same RPMD method but with a very slightly different PES. This variation highlights the sensitivity of *k*_1_ to the subtleties of the PES.

Burke et al.^16^ report a value of *k*_1_ = ∼5 × 10^–11^ cm^3^ molecule^–1^ s^–1^ for reaction R1 at 300 K, based solely on their *ab initio* calculations, but fitting to other experimental parameters of the system they were investigating, raises this to *k*_1_ = ∼10 × 10^–11^ cm^3^ molecule^–1^ s^–1^. Their uncertainty analysis indicated that at 300 K, there was a factor 2–3 uncertainty in *k*_1_; therefore, the increase of approximately a factor 2 to match room-temperature literature values of *k*_1_ = ∼10 × 10^–11^ cm^3^ molecule^–1^ s^–1^ is not unreasonable, but of course that same factor 2–3 uncertainty also encompasses much lower values consistent with our measurements.

Lastly, the initial calculation of Zhang et al.^15^ on the PES for reaction R1 reported a lower value of *k*_1_ = 2.2 × 10^–11^ cm^3^ molecule^–1^ s^–1^. This earlier PES had a much higher barrier for product formation (−2.8 kJ mol^–1^), and therefore this low value is not surprising. Interestingly their calculations predict a very limited temperature dependence. A more recent calculation by the same group in 2018^18^ returns a much lower energy barrier (−12.5 kJ mol^–1^) and a higher rate coefficient at 300 K (*k*_1_ = 6.64 × 10^–11^ cm^3^ molecule^–1^ s^–1^). The focus of the study of both Zhang et al.^15,18^ papers is the role of water in enhancing the rate of reaction R1. In both studies a significant increase in *k*_1_ with water is predicted, consistent with the experimental observations of this work and experiment and theory on other HO_2_ reactions, such as the self-reaction.

The sensitivity of *k*_1_ to the PES (magnitude of well depth, barrier height, and low-frequency vibrations) means that even typical uncertainties in high-level calculations (e.g., <4 kJ mol^–1^ in barrier heights) lead to wide variations in the calculated rate coefficients, at least a factor of 3, whether rate coefficients are calculated using dynamical or statistical approaches. The variation in the calculated value of *k*_1_ using MESMER with input parameters is explored further in the SI, Section S7. Here, we vary the energy, low-frequency vibrations, and imaginary frequency from the calculations of Burke et al. input into MESMER. resulting in variation of *k*_1_ from 2.1 to 10.5 × 10^–11^ cm^3^ molecule^–1^ s^–1^ (see Figure S9). A similar variation is observed if we vary the parameters generated by Method A in a similar fashion, although this variation will be centered around a lower value as our barrier is less submerged than that of Burke et al. Therefore, while we note that most unadjusted calculations give *k*_1,300K_ less than the IUPAC recommendation, but greater than our experimental determination, calculations are unable to definitively resolve the discrepancies in experimental determinations. Calculations do predict the observed significant enhancement with water.

### Comparison with Other Radical–Radical Reactions

Here, we compare the magnitude of *k*_1_ with some other relevant radical–radical “abstractions” from HO_2_. R7, the reaction O + HO_2_, does have a high rate coefficient, *k*_7_ = 6 × 10^–11^ cm^3^ molecule^–1^ s^–1^.
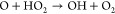
R7In general, OH abstractions occur with higher rate coefficients than the corresponding O reaction, tending to support a high value for *k*_1_; however, isotopic studies^38^ have shown that the mechanism of reaction R7 occurs via HOOO formation and subsequent decomposition to OH and O_2_ and not by a direct abstraction; therefore, direct comparisons of *k*_1_ and *k*_7_ are not appropriate.

H + HO_2_ also has a high rate overall coefficient (*k*_8_ = 8 × 10^–11^ cm^3^ molecule^–1^ s^–1^^13^), but once again analogies with *k*_1_ are limited as the dominant product channel is the formation of 2OH (via decomposition of an H_2_O_2_ intermediate). The abstraction products H_2_ and O_2_, which would be formed in the analogous channel to reaction R1, are a minor channel with *k*_8b_ = 6 × 10^–12^ cm^3^ molecule^–1^ s^–1^.^13^
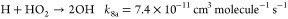
R8a’
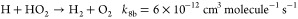
R8bReaction 9: Cl + HO_2_ is the last analogy to be considered. There are two channels available with HCl + O_2_ (reaction R9a) being the dominant channel and the relevant analogy for reaction R1.^39^
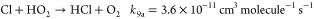
R9a
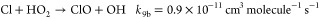
R9b

The reaction has been studied in a flow tube by Hickson and Keyser^39^ who report a total room-temperature rate coefficient of 4.5 × 10^–11^ cm^3^ molecule^–1^ s^–1^. In general, Cl abstraction reactions tend to be faster than the corresponding OH reaction.

We have carried out similar *ab initio* and MESMER calculations on the Cl + HO_2_ reaction R9 similar to those described above for reaction R1. Qualitatively, the PES for R9 is very similar to that for R1 (see SI, Section S8 and Figure S10) with a PRC (−22.7 to −24.6 kJ mol^–1^ depending on the exact methodology) and a submerged barrier (−7.7 to −13.1 kJ mol^–1^) to the HCl + O_2_ products. Our calculations of *k*_9a_, 3–4.2 × 10^–11^ cm^3^ molecule^–1^ s^–1^, are in good agreement with experiment and larger than for the analogous OH reaction, consistent with most Cl/OH systems. The PES is also in excellent agreement with two recent calculations, both in terms of the energies of the stationary points and the kinetics for reaction R9a.^40,41^ Interestingly Zhang et al.^40^ predict no significant enhancement of the rate of reaction 9 in the presence of water.

While there are fewer studies of reaction R9 than reaction R1, there are still a good number (summarized in Table S5), and in contrast to reaction R1, these all seem to be in good agreement on both the absolute rate coefficient (*k*_9_ = 4.2–5.3 × 10^–11^ cm^3^ molecule^–1^ s^–1^) and the branching ratio to channel 9a (∼0.8, *k*_9a_ = 3.2–4.5 × 10^–11^ cm^3^ molecule^–1^ s^–1^). Studies include several flow tube measurements at low pressures and molecular modulation over the pressure range 50–760 Torr. We have no immediate explanation as to why we agree with the flow tube measurements and other studies of R9, but not for reaction R1; Cl radical generation can be carried out in the absence of water, lowering the potential for heterogeneous reactions, this hypothesis requires further evaluation. The relatively tight grouping of the experimental values for *k*_9_ across a range of studies, suggests an absence of significant systematic errors, which may reflect the easier generation of radical reagents and the lack of enhancement by water.

### Implications

Given the importance of the reaction in atmospheric chemistry, it is logical to question why such a difference in the rate coefficient has not led to significant discrepancies in measurement/model comparisons. We suspect that this is primarily due to the water dependence that has been experimentally observed for the first time in this study. Using the observed water dependence from Figure 3: *k*_1,obs_ = 1.02 × 10^–11^ + (2.17 × 10^–28^ × [H_2_O]) cm^3^ molecule^–1^ s^–1^ gives *k*_1,obs_ = 6.5 × 10^–11^ cm^3^ molecule^–1^ s^–1^ for 1% water vapor at atmospheric pressure. This value is similar to the IUPAC recommendation at 300 K.

An important environment to consider the impact of reaction R1 is the upper atmosphere (mesosphere and stratosphere) with typical temperatures in the range of 200–250 K. Water concentrations are low and hence water complexation cannot accelerate *k*_1_. However, most theoretical calculations predict much steeper increases in *k*_1_ with decreasing temperature than are recommended by IUPAC and JPL. Therefore, the significant difference between this work and recommendations at room temperature may be reduced at temperatures relevant to the upper atmosphere. In 2017, Li et al.^1^ tried to resolve the underestimation by atmospheric models (∼50% at 72 km) of observed HO and HO_2_. An overall reduction in *k*_1_ cannot be the simple solution to this problem, as there is better agreement lower in the stratosphere. However, the suggested solution included increasing the rate coefficient of H + O_2_ + M, by up to 310%, significantly beyond the experimental uncertainties of a much simpler system than reaction R1. Further studies are required to assess the temperature dependence of both *k*_1_ and *k*_1,H_2_O_ to assess the impact on stratospheric chemistry.

## Conclusions

The rate coefficient for the reaction of OH with HO_2_ has been determined via two different approaches and over a range of pressures at room temperature. The reported values (*k*_1_ = (1.34 ± 0.14) x 10^–11^ cm^3^ molecule^–1^ s^–1^ from the high-pressure studies, (1.00 ± 0.32) × 10^–11^ cm^3^ molecule^–1^ s^–1^ from the low-pressure studies) are significantly lower than the IUPAC^13^ and JPL recommendations based primarily on the low-pressure flow tube work of Keyser^5^ and on more recent laser flash photolysis studies of Assaf and Fittschen.^3^ Theoretical studies based on a potential energy surface calculated at the RCCSD(T)-F12b/CBS// RCCSD/aug-cc-pVTZ and UCCSD(T)/CBS//UCCSD/aug-cc-pVTZ levels, combined with master equation calculations support a lower value for *k*_1_. However realistic variations in barrier heights and transition state frequencies, give a wide range of calculated rate coefficients.

An enhancement of the rate coefficient due to the complexation of HO_2_ with H_2_O has been predicted by Zhang et al.^15,18^ and is observed for the first time in this work. Such an enhancement can account for some of the experimental discrepancies, but not all. Reaction R1 is of importance in the atmosphere and the water-enhanced rate coefficients measured in this study are similar in magnitude to the IUPAC and JPL recommendations under typical tropospheric conditions, which may explain why field observations have not flagged issues around the magnitude of *k*_1_.

Radical–radical reactions are challenging; while we can speculate on possible reasons for some of the discrepancies in the kinetics, with the exception of studies carried out at high water concentrations, we have no concrete explanation of the differences between this and other studies. We have been careful to explore potential systematic errors, but earlier studies appear to be equally careful. Clearly, further experimental studies, particularly including temperature variation, are warranted.

## Supporting Information Available

Experimental details including schematic diagrams; further details on the ab initio calculations; summary of the experimental data; HO_2_ yield data; details on the potential energy surface and equilibrium constant of the HO_2_–H_2_O complex; further discussion on previous literature on reaction R1; further discussion on the sensitivity of calculated rate coefficients to input parameters; details on the Cl + HO_2_ reaction (R9); and an example MESMER input file (PDF)
