# Supplementary material for: New Measurements and Calculations on the Kinetics of an Old Reaction: OH + HO2 → H2O + O2
Source: JACS Au. 2023 Jun 6;3(6):1684–94. doi: 10.1021/jacsau.3c00110 (PMC10301680; doi:10.1021/jacsau.3c00110)
Supplement: Supplementary file 1 — au3c00110_si_001.pdf [file au3c00110_si_001.pdf]

New Measurements and Calculations on the Kinetics of an Old Reaction: $OH + HO_2 \rightarrow H_2O + O_2$

Thomas H. Speak[1], Mark A. Blitz[1,2*], Diogo J. Medeiros[1], Paul W. Seakins[1*]

[1]*School of Chemistry, University of Leeds, Leeds, LS2 9JT, UK*

[2]*National Centre for Atmospheric Science, University of Leeds, Leeds, LS2 9JT, UK*

Supplementary Information

Contents

S1 – Instrument schematics

S2 – Further details on *ab initio* calculations

S3 – Further example traces on $OH + H_2O_2$ showing variation of rate coefficient with $[OH]_0$ and laser photolysis rate

S4 – HO_2 yield data

S5 – Details on $H_2O{:}HO_2$ complex calculations

S6 – Further discussion on comparison with previous literature on k_1

S7 – Further discussion on the sensitivity of calculated rate coefficients to uncertainties in the calculated input parameters

S8 – Details on the $Cl + HO_2 \rightarrow HCl + O_2$ reaction

S9 – An example MESMER input file.

Unless otherwise stated, reaction numbers refer to the numbering of reactions in the main text, e.g. $OH + HO_2 \rightarrow H_2O + O_2$ is R1 with rate coefficient k_1.

Corresponding Authors

Dr Mark A Blitz, m.blitz@leeds.ac.uk

Prof Paul W Seakins, p.w.seakins@leeds.ac.uk

S1 Instrument Schematics

Figure S1a shows a schematic of the high pressure cell and OH observation cell. Further details can be found in Stone et al.[1] and Speak et al.[2]

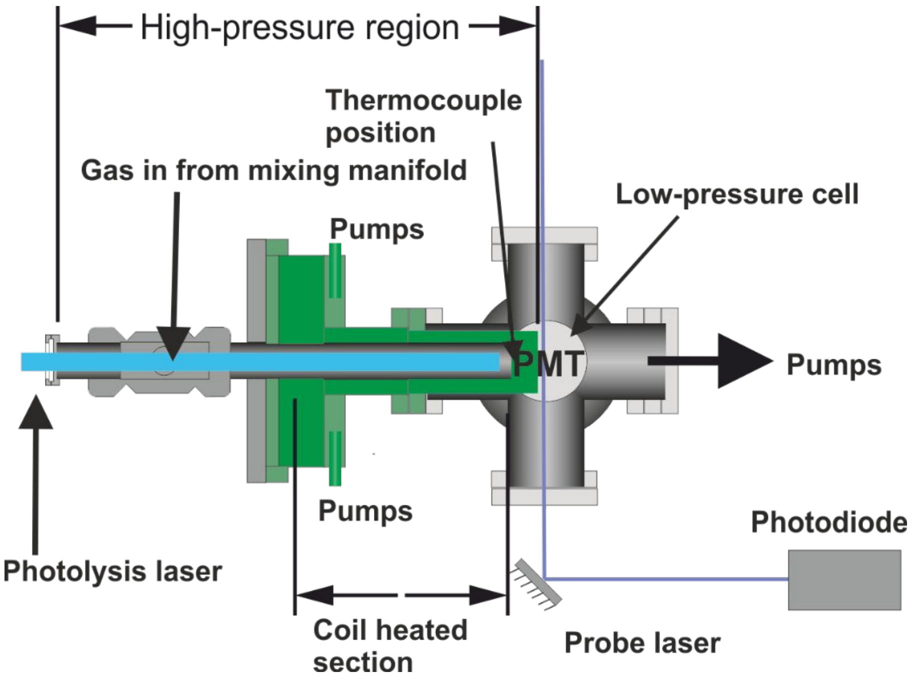

Figure S1a. Schematic of high-pressure reactor. The photolysis laser illuminates the reactor (green, and which can be heated), where the reaction zone is defined by the volume within ~1-2 mm of the pinhole. The OH is detected in the low-pressure cell, blue, within 1-2 cm of the pinhole where the probed gas is jetting.

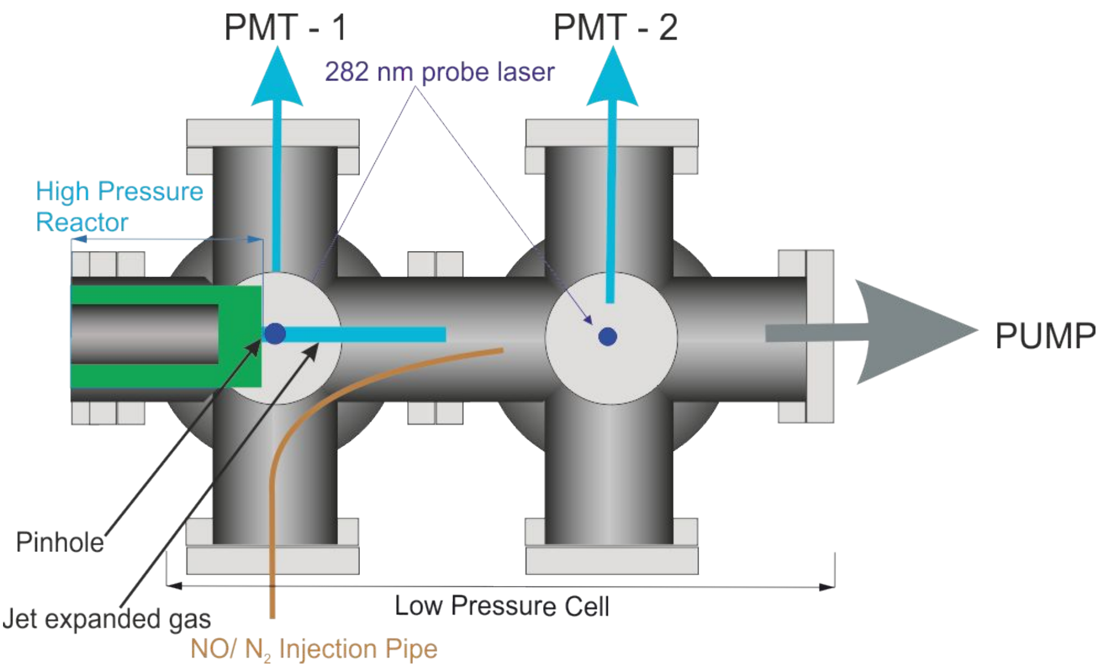

Figure S1b. A schematic of the adapted FAGE cell that allows for simultaneous OH kinetic measurement on the first OH detection axis, PMT-1, and HO_2 yield detection after titration of HO_2 to OH following the breakdown of the 'jet' on a second detection axis, PMT-2.

Figure S2 shows a schematic of the conventional flash photolysis cell. Further details can be found in Onel et al.[3] or Glowacki et al.[4]

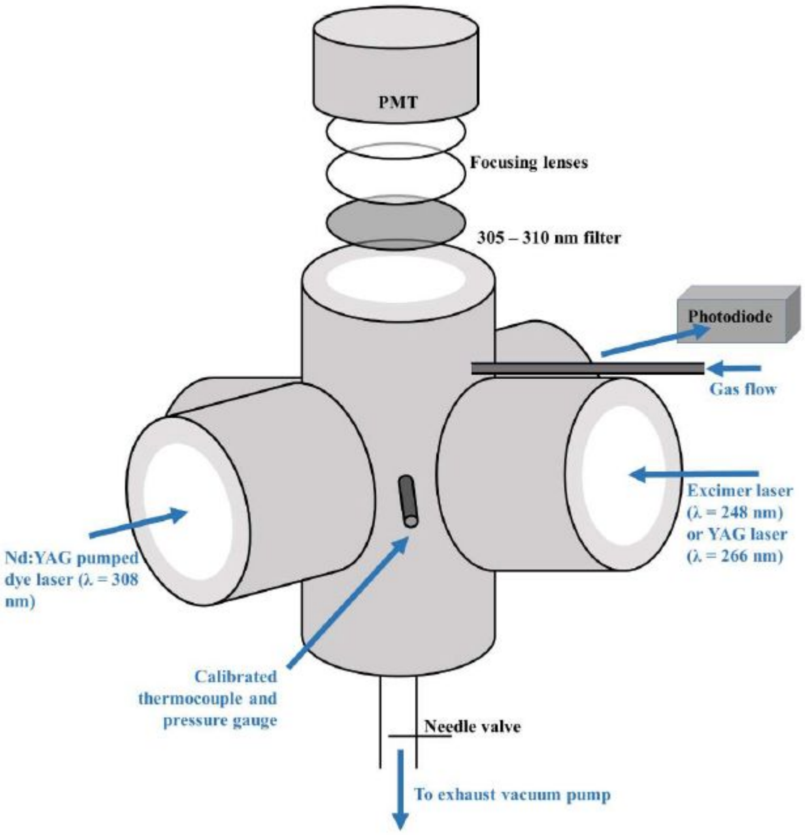

Figure S2. Schematic of low-pressure reactor: the laser beams cross in the centre of the heated, multi-axes cell and defines the reaction zone where the OH is formed and monitored *in situ*, via detecting the fluorescence at right angles to the laser beams.

S2 Further details on *ab initio* calculations

The structures of the transition states found here were obtained using the same methodology for various levels of calculation, by first starting from structures far from the transition state and scanning the breaking and forming bonds. The peak in these scans was used as the input structure for the later transition state optimizations. In addition, transition state structures found with other methods were also used as the input guess structures. Any optimized transition state structures were assessed by IRC jobs to verify if they led to products and the pre reaction complex.

A range of DFT and post HF methods were used to calculate structures and high level single point energy calculations were performed on these structures (CCSD(T)/CBS(aug-cc-pvtz, aug-cc-pvqz, aug-cc-pv5z and jun-cc-pvtz, jun-cc-pvqz, jun-cc-pv5z). The results presented in this paper were for structures calculated with M06-2X using the triple zeta 6-311++g(3df,3pd) and aug-cc-pvtz basis sets carried out in Gaussian. Additional structures were found with UMP2 and the same triple zeta basis sets again in Gaussian and using restricted open shell MP2 in Molpro 2012 with the aug-cc-pvtz basis set. Differences between the structures for the transition states obtained with these different methods led us to evaluate the surface further with a series of further DFT functionals (B2PLYP-D3,

BHandHLYP, wB97XD) and CCSD with the aug-cc-pvtz basis set. The CCSD calculations were performed in Gaussian for UCCSD and in Molpro 2012 for restricted open shell RCCSD. Again the structure and frequencies of the transition states differed with the pre reaction complex being well defined by all methods. Additional, CASSCF(14o,20e)/aug-cc-pvtz calculations were performed in Molpro 2012 and the structure found by this was closest to the structure obtained at the RCCSD/aug-cc-pvtz than that found with UCCSD/aug-cc-pvtz.

The resultant transition state structures could be broadly separated into two groups those with a shorter HO—H bond and more out of plane H-O-O-O dihedral and those with a longer HO—H bond and a flatter dihedral. Further exploration of the surface by using semi relaxed scans of both bond lengths and dihedrals to map out the surface showed that for each method evaluated the surface was not a smooth even surface as is common for many systems but had many troughs and peaks and discontinuities due to the methods utilised here not fully and accurately capturing the real behaviour of the system.

When the different structures were included in MESMER calculations QM rotors were utilised and QM tunnelling was accounted for in these calculations using the Eckhart model as described in the MESMER manual. The different rate coefficients derived from the differing surfaces varied by over an order of magnitude and covered the whole range of previous experimental measurements at 300 K.

Table 2 in the main manuscript shows the variation in the low frequency vibrations in the PRC and TS that are significant in influencing the rate coefficient for the OH + HO_2 reaction as a function of the level of theory. Table S1 gives the complete set of vibrational frequencies.

Table S1 – Full list of the vibrational frequencies of the TS and PRC for various levels of theory

Vibration/cm^{-1}	A	B	C	D	E	F	G
TS imaginary	-2658	-2020	-1387	-1524	-2441	-2521	-3162
TS v1	251	110	205	101	397	395	292
TS v2	520	195	388	164	503	493	566
TS v3	742	458	551	475	693	694	736
TS v4	775	656	857	640	770	773	765
TS v5	1273	1113	1104	1133	1319	1336	1330
TS v6	1410	1451	1310	1520	1387	1403	1992
TS v7	1972	1544	2270	1872	2047	2030	3780
TS v8	3818	3754	3801	3761	3816	3861	4248
PRC v1	148	148	195	206	151	149	178
PRC v2	204	205	217	233	225	213	227
PRC v3	250	257	238	252	307	286	326
PRC v4	422	451	413	350	503	491	499
PRC v5	470	475	517	533	523	508	531
PRC v6	1185	1185	1281	1299	1294	1306	1238
PRC v7	1542	1539	1532	1539	1537	1529	1541
PRC v 8	3582	3575	3509	3535	3474	3517	3477
PRC v9	3700	3697	3656	3680	3705	3752	3696

A=RCCSD(T)-F12b/CBS//RCCSD/aug-cc-pVTZ
B=UCCSD(T)/CBS//UCCSD/aug-cc-pVTZ
C=UCCSD(T)/CBS//UM06-2X/aug-cc-pVTZ
D=UCCSD(T)/CBS//UM06-2X/6-311++g(3df,3pd)
E=UCCSD(T)/CBS//UMP2/aug-cc-pVTZ
F=UCCSD(T)/CBS//UMP2/6-311++g(3df,3pd)
G=D=RCCSD(T)-F12b/CBS//RMP2/aug-cc-pVTZ

S3 Further example traces on OH + H_2O_2 showing variation of rate coefficient with $[OH]_0$ and laser photolysis rate

Figure S3 shows a series of traces in the high pressure system with increasing initial concentration of OH. The pseudo-first order rate coefficients from the individual fits to the traces show an increase of approximately 300 s^{-1} from the lowest to highest $[OH]_0$ in stark contrast to an increase of ~1000 s^{-1} in the fits to the simulated data based on the IUPAC data.

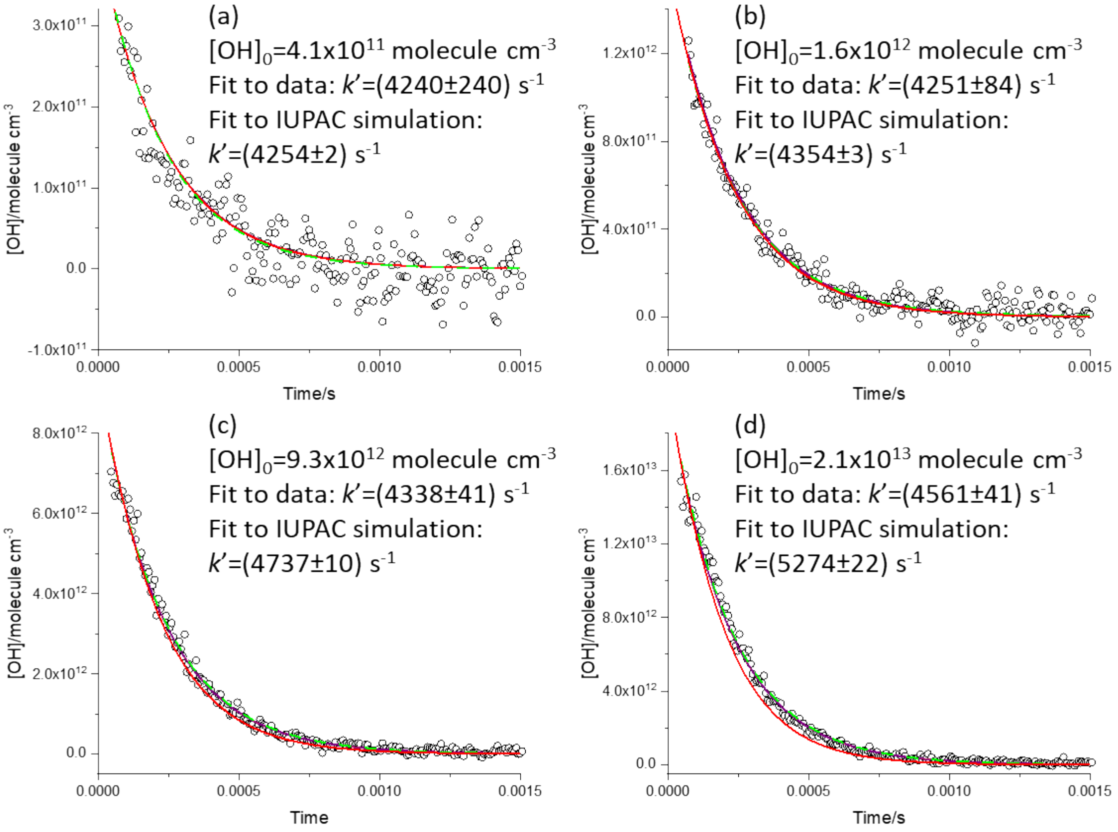

Figure S3. Four example traces with varying laser power in 1800 Torr of N_2 in the high-pressure apparatus at 298 K. These traces differ in their initial OH concentration ranging from a) $[OH]_0 = 4.1 \times 10^{11}$ molecule cm^{-3}, to d) $[OH]_0 = 2.1 \times 10^{13}$ molecule cm^{-3}. (○), data points; (—), fit from global analysis; (—), exponential fit to that single data trace; (—) simulation using $[OH]_0$ and IUPAC rate coefficients.

Each photolysis pulse in the low pressure experiment should photolyse a fresh sample of gas and therefore there should be no secondary chemistry from the photolysis of any reaction products that could alter the observed rate coefficients. This is confirmed by the data shown below in Figure S4 where the reaction of OH with H_2O_2 is followed under identical conditions, with the exception of the laser pulse repetition rates which were reduced from 10 to 2 Hz. The resulting two pseudo-first order rate coefficients are within experimental error, demonstrating a negligible influence of secondary chemistry from product photolysis.

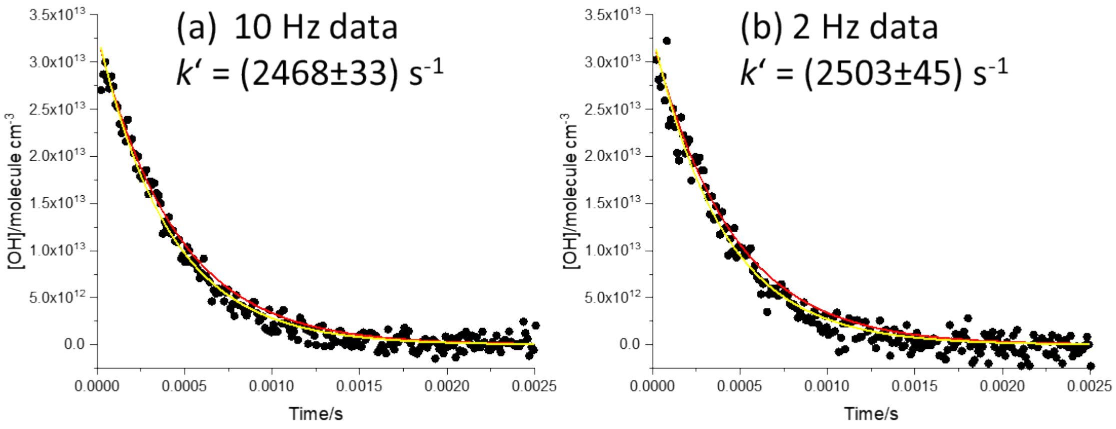

Figure S4. Variation in pseudo-first order rate coefficient as a function of laser repetition rate in the low pressure system. The red line shows the global fit to these particular traces based on the analysis of the full dataset of 47 traces, the yellow line is the exponential fit to the individual trace with the resulting pseudo-first order rate coefficient shown against each trace.

S4 HO_2 yield data

The high pressure system has the capability of observing HO_2 production via titration of HO_2 to OH by NO. The titration reaction occurs in the low pressure observation region following the break up of the jet; transport and titration time means that there is some loss of kinetic fidelity for pseudo-first-order kinetics greater than 3000 s^{-1}, however, the HO_2 yield should not be affected.

Figure S5(a) shows HO_2 traces carried out with identical $[H_2O_2]$ (and hence $[OH]_0$) and $[O_2]$, but with the red points recorded in the presence of 3.27×10^{16} molecule cm^{-3} of methanol. This high concentration of methanol will intercept most OH and lead to the production of HO_2 via the well-characterised reactions:

$OH + H_2O_2 \rightarrow H_2O + HO_2$	$k' = 1.7 \times 10^{-12} \times 1.1 \times 10^{15} = 1870\ s^{-1}$
$OH + CH_3OH \rightarrow H_2O + CH_2OH$	$k' = 9.1 \times 10^{-13} \times 3.27 \times 10^{16} = 29757\ s^{-1}$
$CH_2OH + O_2 \rightarrow HO_2 + HCHO$	$k' = 9.6 \times 10^{-12} \times 6.0 \times 10^{18} = 5.8 \times 10^{8}\ s^{-1}$

Fitting the data gives identical HO_2 yields within experimental error, but Figure S5(b) show a Kintecus[5] simulation, where, with $k_1 = 1.1 \times 10^{-10}$ cm^3 $molecule^{-1}$ s^{-1}, the predicted HO_2 in the absence of methanol should be significantly lower.

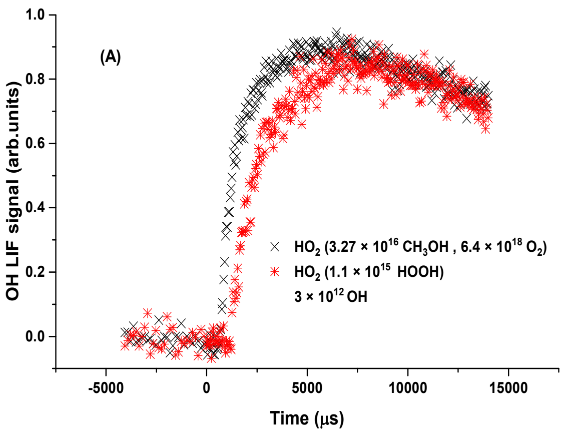

Figure S5(a). A comparison of an HO_2 trace with $[CH_3OH] = 0$ molecule cm^{-3} (red) and $[CH_3OH] = 3.27 \times 10^{16}$ molecule cm^{-3} (black). Both traces were taken with $[O_2] = 6 \times 10^{18}$, $[H_2O_2] = 1.1 \times 10^{15}$ and $[OH]_0 = 3 \times 10^{12}$ molecule cm^{-3}. The assigned HO_2 yield was ≈ 100 %.

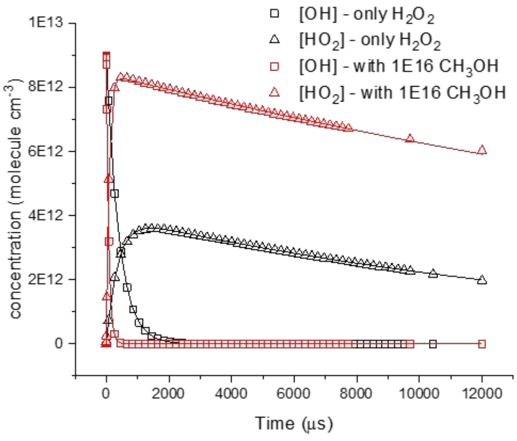

Figure S5(b). A simulation of the expected HO_2 yields for reaction of 9×10^{12} OH with 1×10^{15} H_2O_2 with 6×10^{18} O_2, in the presence and absence of 1×10^{16} methanol. Where the removal of OH by reaction with the HO_2, OH were included, and accounting for the loss of HO_2 via reaction with HO_2, OH, CH_3O and diffusion.

Similar experiments were recorded over a range of conditions and the results are tabulated in Table S2.

Table S2. Relative HO_2 yields for OH and H_2O_2 compared to the yield from the calibration reaction of OH and methanol in high oxygen (6.4×10^{18} cm^{-3}) for varied $[OH]_0$.

$[OH]_0$ / molecule cm^{-3}	HO_2 yield			
	1	2	3	average
1.1×10^{11}	92	106	98	98 ± 7
3.0×10^{12}	97	98	101	99 ± 2
2.6×10^{13}	98	97	103	98 ± 3

Within experimental error, the yields with and without added methanol are identical suggesting limited interference from reaction R1 on the kinetics of the OH + H_2O_2 reaction. Figure S6 explores this issue via simulations over a wide range of values of k_1 for the highest initial concentration of OH used (2.6×10^{13} molecule cm^{-3}). The observed agreement in the HO_2 yield between experiments with and without methanol limits k_1 to less than $\sim 1 \times 10^{-11}$ cm^3 molecule^{-1} s^{-1}.

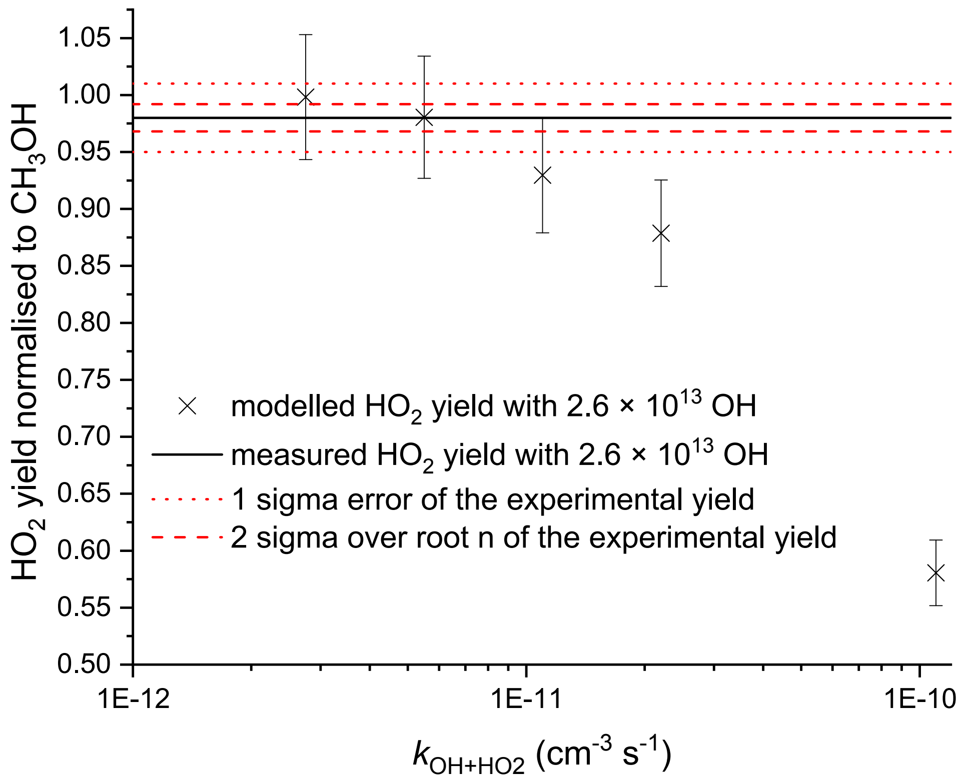

Figure S6. Relative HO_2 yield of OH and H_2O_2 (2.7×10^{15} molecule cm^{-3}) compared to OH and CH_3OH ($[CH_3OH] = 2.5 \times 10^{16}$ molecule cm^{-3} with $[O_2] = 6.4 \times 10^{18}$ molecule cm^{-3}) and $[OH]_0 = 2.6 \times 10^{13}$ molecule cm^{-3}.The experimental observed value is shown as the black line with the modelled output shown as black points.

S5 The H_2O:HO_2 Complex

Key to calculations on the water mediated reaction of OH + HO_2 is ensuring that the initial H_2O:HO_2 complex is correctly calculated. Figure S7 shows the PES and the structure of the complex calculated at the CCSD(T)/CBS//CCSD/6-311++g(3df, 3dp) level of theory.[6]

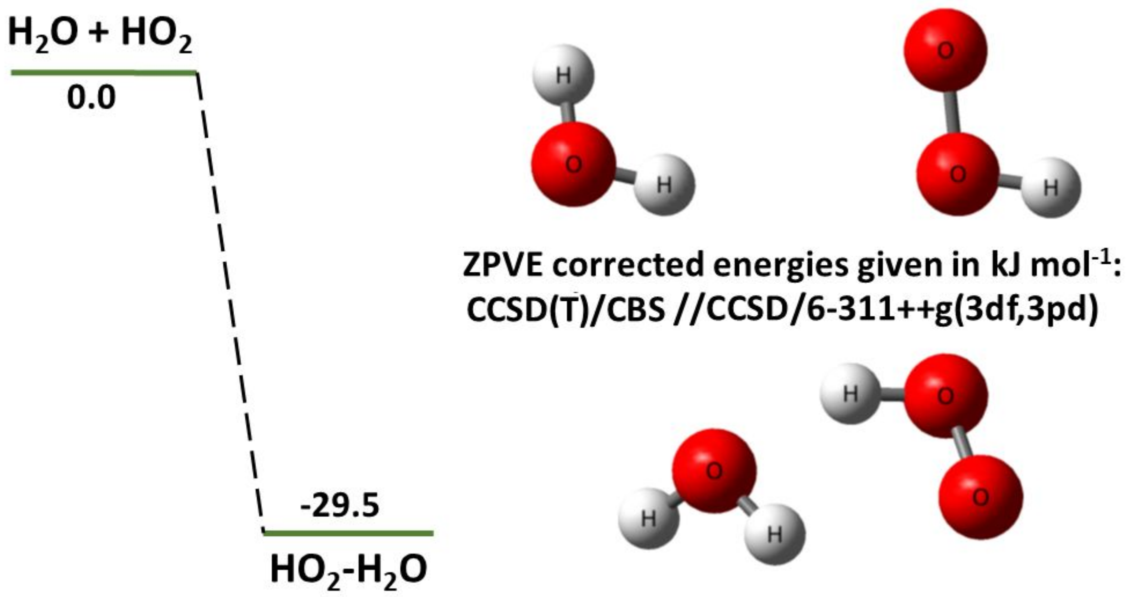

Figure S7. Potential energy surface for the formation of the H_2O:HO_2 complex calculated at the CCSD(T)/CBS//CCSD/6-311++g(3df,3pd) level using Gaussian 09.[6]

The equilibrium constants calculated from this PES are in excellent agreement with those from Kanno et al.[7] as shown in Table S3.

Table S3. Comparison of experimental values (1) and values taken from the temperature dependent parameterization (2) for the equilibrium constant for the formation of HO_2-H_2O, from Kanno *et al*.[7] with computational (from MESMER[8]) values generated in this work.

Temperature (K)	Kanno et al. 2006		MESMER
	K_{eq} (molecule^{-1} cm^3) (1)	K_{eq} (molecule^{-1} cm^3) *(2)*	K_{eq} (molecule^{-1} cm^3)
350	1.10E-19	9.01E-20	9.86E-20
325	2.10E-19	1.89E-19	2.11E-19
298		4.85E-19	5.15E-19
297	5.20E-19	5.04E-19	5.36E-19
288.2		7.16E-19	8.47E-19
275.2		1.25E-18	1.51E-18
273	1.30E-18	1.39E-18	1.67E-18
262.2		2.33E-18	2.87E-18
250	6.00E-18	4.42E-18	5.62E-18
249.3		4.59E-18	5.86E-18
236.3		9.85E-18	1.30E-17
230		1.47E-17	1.99E-17
223.3		2.31E-17	3.21E-17
216.7		3.72E-17	5.29E-17

The HO_2:H_2O equilibrium constant can now be used to calculate the concentration of complexes for any [H_2O], assuming that equilibrium is achieved. Figure S8 shows an analogous plot to Figure 3 of the main text, where now the pseudo-first order rate coefficient is plotted against [HO_2:H_2O].

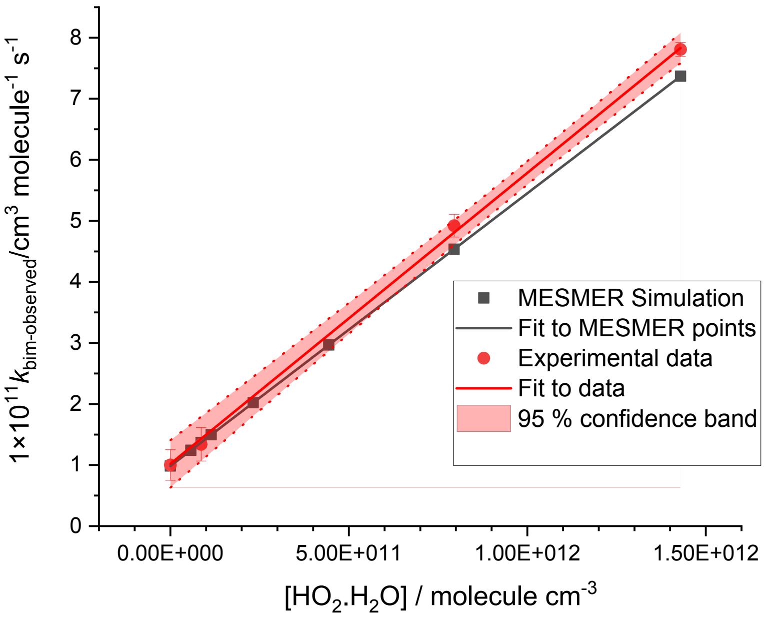

Figure S8. A plot of the observed bimolecular rate coefficient as a function of the [HO_2:H_2O]. [HO_2:H_2O] calculated from the equilibrium constant discussed above.

S6 Further comparison with previous literature on k_1
The main reactions contributing to the IUPAC evaluation of reaction 1 have been considered in the main text, however, there have been a number of other studies of reaction 1 which are considered below.

Cox et al., 1981[9], k_1 = (9.9 ± 2.5) × 10^{-11} cm^3 $molecule^{-1}$ s^{-1} – Molecular modulation methods were used, monitoring the absorption of HO_2 over a 1 s timescale. OH and HO_2 were generated via the photolysis of O_3 in the presence of up to 10 Torr of water where Δk_1, the change in the OH + HO_2 reaction due to gas phase complexation of HO_2 and H_2O could be up to to 8 × 10^{-11} cm^3 $molecule^{-1}$ s^{-1}. The reported value of k_1 is again dependent on the HO_2 recombination rate coefficient, itself water dependent, and a complex kinetic model.

De More, 1982[10], k_1 = (12 ± 4) × 10^{-11} cm^3 $molecule^{-1}$ s^{-1} – These studies were carried out via the continuous photolysis of water vapour (1-5 Torr) at 185 nm and monitoring the steady-state concentration of OH via laser induced fluorescence. The parameter determined is the ratio $\frac{k_1}{(k_{HO2\ recomb})^{0.5}}$, where $k_{HO2\ recomb}$ is the rate coefficient for the reaction of HO_2. k_1 was reported to range from 5.8 × 10^{-11} cm^3 $molecule^{-1}$ s^{-1} at 75 Torr total pressure and 1 Torr of water to 1.2 × 10^{-10} cm^3 $molecule^{-1}$ s^{-1} at 730 Torr total pressure and 5 Torr of water. According to Figure 3 in the main text, the presence of 5 Torr of water should produce a significant enhancement (Δk_1 = 4 × 10^{-11} cm^3 $molecule^{-1}$ s^{-1}) in the rate coefficient. Data were interpreted in terms of a potential pressure dependence for reaction 1, but it is likely that the data were influenced by the water dependence of both the OH + HO_2 and the HO_2 recombination reactions. Reactions were carried out over several seconds and hence there is also a potential for heterogeneous effects.

Braun et al., 1982[11], k_1 = (11.0 ± 3.0) × 10^{-11} cm^3 $molecule^{-1}$ s^{-1} – Again in this study VUV photolysis of water was used to generate OH and HO_2, however, in this case via a time resolved process. OH was detected via resonance fluorescence and k_1 was extracted from a complex analysis varying the amount of O_2 present and hence the amount of H from the initial photolysis which is converted to HO_2. Concentrations of water vapour used are significantly lower than in the De More study, typically ~0.4 Torr and hence will have a limited enhancement (<1 × 10^{-11} cm^3 $molecule^{-1}$ s^{-1}) on the observed value of k_1.

Dransfeld and Wagner, 1987[12], k_1 = (6.0 ± 1.5) × 10^{-11} cm^3 $molecule^{-1}$ s^{-1} – Dransfeld and Wagner generated ^{18}OH and monitored via laser magnetic resonance in a flow tube with excess $H^{16}O_2$. They saw a factor two enhancement in the rate coefficient in comparison to the ^{16}OH reaction which they attributed to the reaction that occurs on the singlet potential surface where $H^{18}O^{16}O^{16}OH$ will be formed. Approximately 50% of the time, in which $H^{18}O^{16}O^{16}OH$ is formed, ^{18}OH will be lost when the complex re-dissociates. Most relevant for our study is the rate coefficient for the ^{16}OH which is (6.0 ± 1.5) × 10^{-11} cm^3 $molecule^{-1}$ s^{-1}. As with all other studies, there is a careful analysis of potential errors. Water is used in the generation of OH, but at concentrations that will provide an insignificant enhancement of k_1. We have no explanation of the difference between this work and Dransfeld and Wagner, but we do note that pseudo-first-order rate coefficients are around 30 s^{-1}, so there is potential for heterogeneous chemistry to contribute to radical loss processes.

In summary the four previous studies discussed above provide results in stark contrast to the direct measurements of this work. Water dependence could account for the differences in the DeMore and Cox studies as water vapour concentrations were significant in these studies. Although water was used as a precursor in the studies of Dransfeld and Wagner and Braun et al., water was present at such low concentrations that it could not have contributed through gas phase complexation of HO_2, but reactions were carried out under conditions where heterogeneous processes could have contributed. In all studies, k_1 has been extracted from complex and from relatively slow chemistry enhancing the potential for secondary chemistry or other systematic issues. Of course, as stated in the main text, we cannot rule out the potential for unknown systematic errors in our work.

S7 Further discussion on the sensitivity of calculated rate coefficients to uncertainties in the calculated input parameters

Table 2 in the main manuscript shows the variation in barrier heights, transition state frequencies and imaginary frequency for a range of ab initio calculations. The resulting calculations of k_1 from MESMER give values ranging from (0.24 - 9.2) × 10^{-11} cm^3 $molecule^{-1}$ s^{-1}. This wide range arises from simultaneous variation in several parameters important for the calculation. Here we report a study looking systematically at the variation of barrier height, low frequency and imaginary vibrations, and grain size used in the MESMER master equation calculation. The results presented here use the energies and frequencies calculated using the CASPT2 calculations of Burke et al. although similar results were obtained varying the same parameters from the Method A calculation. The objective here is not to examine the absolute values (we know these are variable), but rather to systematically examine the variation of the MESMER calculated value of k_1 with these parameters.

i) *Grain size* – in a master equation, energies of the species are divided into grains. The smaller the grain size, the more accurate, but also, more expensive the calculation is. Table S4a shows the variation in k_1 with grain size. Our calculations typically use a 50 cm^{-1} grain size which is a good compromise between precision and computational cost.

Table S4a – Variation of k_1 with grain size

Grain size/cm^{-1}	k_1/cm^3 $molecule^{-1}$ s^{-1}
200	7.34×10^{-11}
100	7.05×10^{-11}
50	6.95×10^{-11}
25	6.93×10^{-11}
12.5	6.93×10^{-11}

ii) Barrier height – Burke et al. calculated a barrier height of -11.42 kJ mol^{-1}, somewhat lower than the -5.1 kJ mol^{-1} from our Method A calculation. Table S4b shows the near linear dependence of k_1 with barrier height.

Table S4b – Variation of k_1 with barrier height

Barrier height/kJ mol^{-1}	k_1/cm^3 $molecule^{-1}$ s^{-1}
-11.42	6.95×10^{-11}
-5.72	3.35×10^{-11}
-2.86	2.08×10^{-11}

iii) Low frequency vibrations in the transition state – Densities of states and state counts required for RRKM calculations are strongly influenced by the lowest frequency vibrations. Indeed, whether a motion is treated as a vibration or a hindered rotor, can have a significant effect. Table S4c shows the variation in k_1 as the lowest vibrational frequency for the transition state calculated by Burke et al. (121 cm^{-1}) is either halved or doubled. As would be expected, decreasing the vibrational frequency increases the number of states accessible in the transition state and hence increases $k_{1.}$

Table S4c – Variation of k_1 with low frequency vibrations

Lowest vibrational frequency/cm^{-1}	k_1/cm^3 $molecule^{-1}$ s^{-1}
60	10.5×10^{-11}
121	6.95×10^{-11}
242	4.62×10^{-11}

iv) – Imaginary frequency – Table S4d shows the variation in k_1 is the imaginary frequency. The range has been chosen deliberately to be so wide, reflecting the magnitude of the variation in the imaginary frequency as shown in Table 2. There is a significant effect, with the reaction being enhanced by a higher imaginary frequency.

Table S4d – Variation of k_1 with imaginary frequency

Imaginary frequency/cm^{-1}	k_1/cm^3 molecule^{-1} s^{-1}
205	4.55 x 10^{-11}
2056	6.95 x 10^{-11}
4056	8.73 x 10^{-11}

In summary, individually varying the barrier height, low frequency vibrations and imaginary frequency of the Burke et al. calculations input into MESMER can vary k_1 from 2.1 – 10.5 x 10^{-11} cm^3 molecule^{-1} s^{-1}. Figure S9 summarizes the impact of varying the lowest frequency vibration and imaginary frequency on k_1. A similar variation is observed if we vary the parameters generated by the Method A in a similar fashion, although this variation will be centred around a lower value as our barrier is lower than that of Burke et al. Essentially, anything that increases the state count at the transition state (a lower barrier height or lower vibrational frequencies in the transition state) will increase k_1. The sensitivity of the MESMER calculations to the barrier height and transition state frequencies mean the uncertainties in these parameters translate into calculated rate coefficients which can span all experimental determinations of k_1.

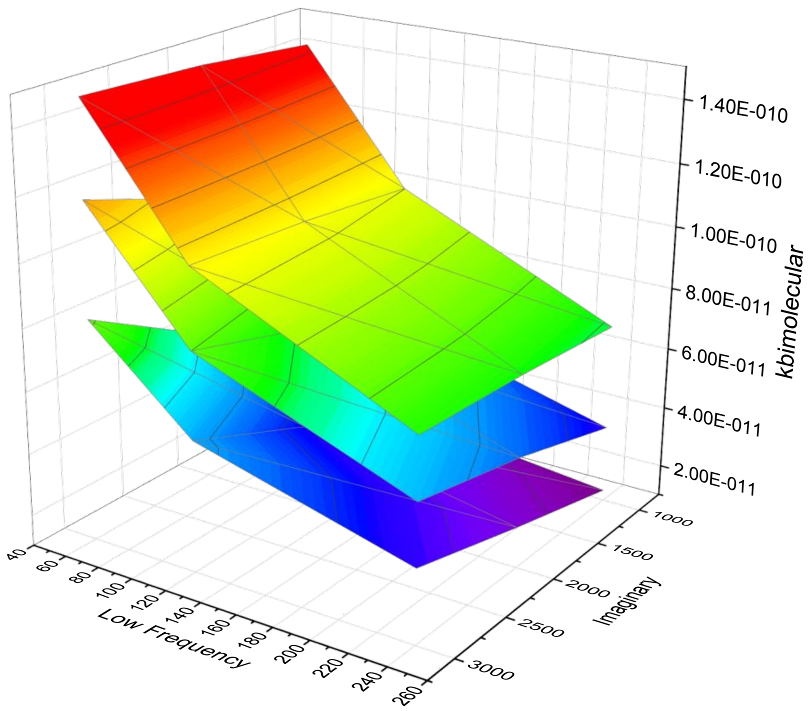

Figure S9. Plot of the variation of k_1 with the lowest frequency of the transition state, the imaginary frequency and for three barrier heights (from the top: -11.42, -5.71 and -2.86 kJ mol^{-1}) calculated via MESMER.

S8 Details on the Cl + HO_2 → HCl + O_2 reaction

Figure S10 shows the triplet potential energy surface for the reaction of Cl + HO_2. Qualitatively the PES is similar to the OH + HO_2 reaction with the formation of a pre-reaction complex and a submerged barrier for abstraction. The reaction is known to also produce OH + ClO, but as can be seen from Figure S10, this will occur on the singlet surface via the formation of an HOOCl intermediate, analogous to the null reaction via HOOOH on the singlet surface for the OH + HO_2 system.

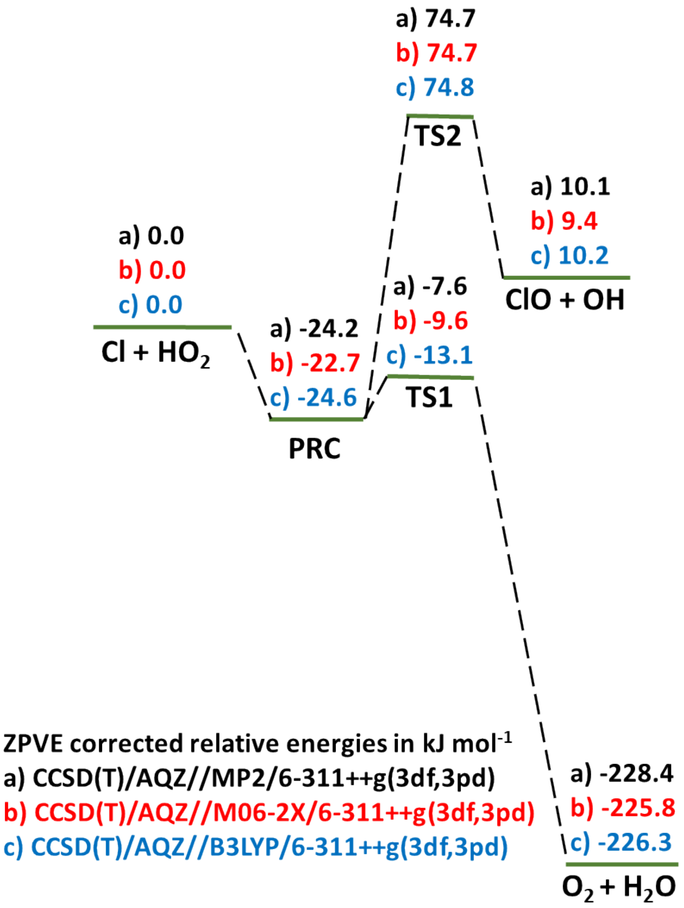

Figure S10. Triplet potential energy surface for the Cl + HO_2 reaction carried out using Gaussian 09 at various levels of theory.

Examples of previous studies on the Cl + HO_2 reaction are given in the table below.

$$Cl + HO_2 \rightarrow HCl + O_2 \qquad \text{R9a}$$

$$Cl + HO_2 \rightarrow ClO + OH \qquad \text{R9b}$$

Table S5. Examples of experimental studies on the Cl + HO_2 reaction

Overall Reaction: Cl + $HO_2 \rightarrow$ products		
Author	Date	$10^{11} \times k_9$ /cm^3 molecule^{-1} s^{-1}
Hickson and Keyser[13]	2005	4.5 ± 0.4
Riffault et al.[14]	2001	4.4 ± 0.6
Dobis and Benson[15]	1993	4.45 ± 0.06
Lee and Howard[16]	1982	4.2 ± 0.7
Cl + $HO_2 \rightarrow$ HCl + O_2		
Hickson and Keyser[13]	2005	3.7 ± 0.4[a]
Riffault et al.[14]	2001	3.5 ± 0.7
Cattell and Cox[17]	1986	4.4 ± 2.2
Lee and Howard[16]	1982	3.2 ± 0.7

a – rate coefficients for this section of the table refer to k_{9a}

S9 An example MESMER input file


```
<?xml version="1.0" encoding="utf-8" ?>
<?xml-stylesheet type='text/xsl' href='../../mesmer2.xsl' media='other'?>
<?xml-stylesheet type='text/xsl' href='../../mesmer1.xsl' media='screen'?>
<me:mesmer xmlns="http://www.xml-cml.org/schema"
        xmlns:me="http://www.chem.leeds.ac.uk/mesmer"
        xmlns:xsi="http://www.w3.org/2001/XMLSchema-instance">
 <me:title> OH + HO2 - triplet RCCSD/aug-cc-pvtz</me:title>


 <moleculeList>

  <molecule id="OH-2">
 <atomArray>
  <atom id="a1" elementType="O" x3="0.000000" y3="0.000000" z3="0.107775"/>
  <atom id="a2" elementType="H" x3="0.000000" y3="0.000000" z3="-0.862899"/>
 </atomArray>
 <bondArray>
  <bond atomRefs2="a2 a1" order="1"/>
 </bondArray>
      <propertyList>
         <property dictRef="me:ZPE">
            <scalar units="kJ/mol">0</scalar>
         </property>
         <property dictRef="me:symmetryNumber">
            <scalar>1</scalar>
         </property>
         <property dictRef="me:frequenciesScaleFactor">
            <scalar>0.953</scalar>
         </property>
       <property title="Vibrational Frequencies" dictRef="me:vibFreqs">
  <array units="cm-1">3756.56 </array>
 </property>
 <property title="Rotational Constants" dictRef="me:rotConsts">
   <array units="cm-1">18.87361774 18.87361774 0 </array>
 </property>
         <property dictRef="me:MW">
            <scalar units="amu">17.0027396518</scalar>
         </property>
         <property dictRef="me:spinMultiplicity">
            <scalar>2.00</scalar>
```

```
        </property>
                         <property dictRef="me:sigma">
       <scalar>4.74</scalar>
      </property>
      <property dictRef="me:epsilon">
       <scalar>233.0</scalar>
      </property>
      </propertyList>
                         <!-- <me:DOSCMethod>ClassicalRotors</me:DOSCMethod> -->
                        <me:DOSCMethod>QMRotors</me:DOSCMethod>
                         <me:energyTransferModel xsi:type="me:ExponentialDown">

                         <me:deltaEDown bathGas="N2" units="cm-1"  >250</me:deltaEDown>
                          <me:deltaEDownTExponent bathGas="N2" referenceTemperature="298"
>0.25</me:deltaEDownTExponent>

     </me:energyTransferModel>
    </molecule>

            <molecule id="HO2-2">
 <atomArray>
  <atom id="a1" elementType="O" x3="-0.066174" y3="0.000000" z3="-0.632730"/>
  <atom id="a2" elementType="H" x3="0.856745" y3="0.000000" z3="-0.931765"/>
  <atom id="a3" elementType="O" x3="0.012191" y3="0.000000" z3="0.691439"/>
 </atomArray>
 <bondArray>
  <bond atomRefs2="a2 a1" order="1"/>
  <bond atomRefs2="a1 a3" order="1"/>
 </bondArray>
      <propertyList>
        <property dictRef="me:ZPE">
          <scalar units="kJ/mol">0</scalar>
        </property>
 <property title="Vibrational Frequencies" dictRef="me:vibFreqs">
  <array units="cm-1">1162.97 1464.78 3700.63 </array>
 </property>
  <property title="Rotational Constants" dictRef="me:rotConsts">
      <array units="cm-1">1.129453455 1.071165718 20.75611694</array>
  </property>
        <property dictRef="me:symmetryNumber">
          <scalar>1</scalar>
        </property>
        <property dictRef="me:frequenciesScaleFactor">
          <scalar>0.953</scalar>
        </property>
        <property dictRef="me:MW">
          <scalar units="amu">32.9976542714</scalar>
        </property>
        <property dictRef="me:spinMultiplicity">
          <scalar>2</scalar>
        </property>
      </propertyList>
                         <!-- <me:DOSCMethod>ClassicalRotors</me:DOSCMethod> -->
                        <me:DOSCMethod>QMRotors</me:DOSCMethod>

                         <me:energyTransferModel xsi:type="me:ExponentialDown">
                        <me:deltaEDown bathGas="N2" units="cm-1"  >250</me:deltaEDown>
                        <me:deltaEDownTExponent bathGas="N2" referenceTemperature="298"
>0.25</me:deltaEDownTExponent>
     </me:energyTransferModel>

   </molecule>
```

```
<molecule id="PRC-2">
 <atomArray>
<atom id="a1" elementType="O" x3="-0.010658" y3="0.000000" z3="-1.814874"/>
<atom id="a2" elementType="H" x3="0.820033" y3="-0.000001" z3="-1.303455"/>
<atom id="a3" elementType="O" x3="-0.654907" y3="0.000000" z3="0.923666"/>
<atom id="a4" elementType="H" x3="-0.849509" y3="0.000000" z3="-0.034478"/>
<atom id="a5" elementType="O" x3="0.667421" y3="0.000000" z3="0.975510"/>
</atomArray>
<bondArray>
<bond id="b1" atomRefs2="a1 a2" order="1"/>
<bond id="b2" atomRefs2="a4 a3" order="1"/>
<bond id="b3" atomRefs2="a3 a5" order="1"/>
<!-- H bonded to OH -->
<bond id="b4" atomRefs2="a1 a4" order="1"/>
</bondArray>
  <propertyList>
    <property dictRef="me:ZPE">
                <scalar units="kJ/mol">-20.99547626</scalar>
    </property>
    <property dictRef="me:symmetryNumber">
      <scalar>1</scalar>
    </property>
    <property dictRef="me:frequenciesScaleFactor">
      <scalar>0.953</scalar>
    </property>
 <property title="Vibrational Frequencies" dictRef="me:vibFreqs">
<array units="cm-1">147.66 204.02 249.71 421.67 470.47 1185.17 1542.00 3582.04 3700.02 </array>
</property>
<property title="Rotational Constants" dictRef="me:rotConsts">
      <array units="cm-1">0.202459219 0.170872223 1.095215945</array>
</property>
    <property dictRef="me:MW">
      <scalar units="amu">50.0003939233</scalar>
    </property>
    <property dictRef="me:spinMultiplicity">
      <scalar>3</scalar>
    </property>

          <property dictRef="me:sigma">
   <scalar>5.58</scalar>
   </property>
      <property dictRef="me:epsilon" >
        <scalar>291</scalar>
      </property>

  </propertyList>

          <!-- <me:DOSCMethod>ClassicalRotors</me:DOSCMethod> -->
          <me:DOSCMethod>QMRotors</me:DOSCMethod>
          <me:energyTransferModel xsi:type="me:ExponentialDown">
            <me:deltaEDown bathGas="N2" units="cm-1"  >250</me:deltaEDown>
            <me:deltaEDownTExponent bathGas="N2" referenceTemperature="298"
>0.25</me:deltaEDownTExponent>
   </me:energyTransferModel>

      <me:ExtraDOSCMethod xsi:type="me:HinderedRotorQM1D">
  <me:bondRef>b4</me:bondRef>
  <me:HinderedRotorPotential format="analytical" units="kJ/mol">
   <me:PotentialPoint index="0"   coefficient="24.6718417415513"/>
   <me:PotentialPoint index="3"  coefficient="-24.6718417415513"/>
  </me:HinderedRotorPotential>
  <me:periodicity>1</me:periodicity>
 </me:ExtraDOSCMethod>
```

```
  </molecule>

        <molecule id="water-2">
 <atomArray>
  <atom id="a1" elementType="O" x3="0.000000" y3="0.000000" z3="-0.065769"/>
  <atom id="a2" elementType="H" x3="0.000000" y3="0.757628" z3="0.521904"/>
  <atom id="a3" elementType="H" x3="0.000000" y3="-0.757628" z3="0.521904"/>
 </atomArray>
 <bondArray>
  <bond atomRefs2="a1 a3" order="1"/>
  <bond atomRefs2="a1 a2" order="1"/>
 </bondArray>
      <propertyList>
        <property dictRef="me:ZPE">
                    <!-- Fraction of energy difference that is water -->
                        <scalar units="kJ/mol">-98.27454168</scalar>
        </property>
        <property dictRef="me:symmetryNumber">
          <scalar>1</scalar>
        </property>
        <property dictRef="me:frequenciesScaleFactor">
          <scalar>0.953</scalar>
        </property>
 <property title="Vibrational Frequencies" dictRef="me:vibFreqs">
  <array units="cm-1">1659.24 3853.04 3959.06 </array>
 </property>
  <property title="Rotational Constants" dictRef="me:rotConsts">
   <array units="cm-1">9.496181378 27.2680941 14.57034052</array>
  </property>
        <property dictRef="me:MW">
          <scalar units="amu">18.0105646841</scalar>
        </property>
        <property dictRef="me:spinMultiplicity">
          <scalar>1</scalar>
        </property>
                    <property dictRef="me:deltaEDown">
       <scalar units="cm-1">100.0</scalar>
      </property>
      </propertyList>
                <!-- <me:DOSCMethod>ClassicalRotors</me:DOSCMethod> -->
                <me:DOSCMethod>QMRotors</me:DOSCMethod>
  </molecule>

<molecule id="O2-t-2">
 <atomArray>
  <atom id="a1" elementType="O" x3="0.000000" y3="0.000000" z3="-0.599839"/>
  <atom id="a2" elementType="O" x3="0.000000" y3="0.000000" z3="0.599839"/>
 </atomArray>
 <bondArray>
  <bond atomRefs2="a1 a2" order="2"/>
 </bondArray>
      <propertyList>
        <property dictRef="me:ZPE">
          <scalar units="kJ/mol">-193.3141335</scalar>
        </property>
 <property title="Vibrational Frequencies" dictRef="me:vibFreqs">
  <array units="cm-1">1671.83 </array>
 </property>
  <property title="Rotational Constants" dictRef="me:rotConsts">
   <!-- <array units="GHz">43.90728447 43.90729926 0 </array> -->
   <array units="cm-1">1.464589362 1.464589855 0</array>
  </property>
        <property dictRef="me:symmetryNumber">
```

```
        <scalar>2</scalar>
      </property>
      <property dictRef="me:frequenciesScaleFactor">
        <scalar>0.953</scalar>
      </property>
      <property dictRef="me:MW">
        <scalar units="amu">31.9898292392</scalar>
      </property>
      <property dictRef="me:spinMultiplicity">
        <scalar>3</scalar>
      </property>
                                  <property dictRef="me:epsilon">
     <scalar>473.17</scalar>
    </property>
    <property dictRef="me:sigma">
     <scalar>5.09</scalar>
    </property>
    <property dictRef="me:deltaEDown">
     <scalar units="cm-1">125.0</scalar>
    </property>
                          </propertyList>
                          <!-- <me:DOSCMethod>ClassicalRotors</me:DOSCMethod> -->
                         <me:DOSCMethod>QMRotors</me:DOSCMethod>
  </molecule>

<molecule id="TS-abst-1-t-2">
<!-- TS ab 8 -->
 <atomArray>
  <atom id="a1" elementType="O" x3="-0.060086" y3="-0.018311" z3="-1.398458"/>
  <atom id="a2" elementType="H" x3="0.831568" y3="0.181396" z3="-1.720472"/>
  <atom id="a3" elementType="O" x3="0.011776" y3="-0.613652" z3="0.809780"/>
  <atom id="a4" elementType="H" x3="0.007557" y3="-0.716517" z3="-0.309381"/>
  <atom id="a5" elementType="O" x3="-0.004563" y3="0.665681" z3="0.716578"/>
 </atomArray>
 <bondArray>
  <bond id="b1" atomRefs2="a2 a1" order="1"/>
  <bond id="b2" atomRefs2="a4 a3" order="1"/>
  <bond id="b3" atomRefs2="a5 a3" order="1"/>
  <bond id="b4" atomRefs2="a1 a4" order="1"/>
 </bondArray>
       <propertyList>
          <property dictRef="me:ZPE">
                                  <scalar units="kJ/mol">-5.123496531</scalar>
          </property>
          <property dictRef="me:symmetryNumber">
            <scalar>1</scalar>
          </property>

                                  <!-- <property dictRef="me:TSOpticalSymmetryNumber"> -->
                                    <!-- <scalar>2</scalar> -->
          <!-- </property> -->

         <property dictRef="me:frequenciesScaleFactor">
            <scalar>0.953</scalar>
         </property>
 <property title="Vibrational Frequencies" dictRef="me:vibFreqs">
  <!-- <array units="cm-1">250.78 520.22 741.55 775.42 1273.27 1409.56 1971.78 3817.99 </array> -->
  <!-- removed the second frequency as the hindered rotor -->
    <array units="cm-1">250.78 741.55 775.42 1273.27 1409.56 1971.78 3817.99 </array>
 </property>
  <property title="ImaginaryFrequency" dictRef="me:imFreqs">
   <scalar units="cm-1">2657.89 </scalar>
   </property>
```

```
&lt;property title="Rotational Constants" dictRef="me:rotConsts"&gt;
  &lt;array units="cm-1"&gt;0.252626901 0.313224593 1.168700143 &lt;/array&gt;
&lt;/property&gt;
        &lt;property dictRef="me:MW"&gt;
          &lt;scalar units="amu"&gt;50.0003939233&lt;/scalar&gt;
        &lt;/property&gt;
                                          &lt;property dictRef="me:deltaEDown"&gt;
      &lt;scalar units="cm-1"&gt;125.0&lt;/scalar&gt;
    &lt;/property&gt;
        &lt;property dictRef="me:spinMultiplicity"&gt;
           &lt;scalar&gt;3&lt;/scalar&gt;
        &lt;/property&gt;
     &lt;/propertyList&gt;
                            &lt;!-- &lt;me:DOSCMethod&gt;ClassicalRotors&lt;/me:DOSCMethod&gt; --&gt;
                           &lt;me:DOSCMethod&gt;QMRotors&lt;/me:DOSCMethod&gt;
                            &lt;me:ExtraDOSCMethod xsi:type="me:HinderedRotorQM1D"&gt;
    &lt;me:bondRef&gt;b4&lt;/me:bondRef&gt;
    &lt;me:HinderedRotorPotential format="analytical" units="kJ/mol"&gt;
      &lt;me:PotentialPoint index="0"   coefficient="9.414"/&gt;
      &lt;me:PotentialPoint index="3"  coefficient="-9.414"/&gt;
    &lt;/me:HinderedRotorPotential&gt;
    &lt;me:periodicity&gt;1&lt;/me:periodicity&gt;
  &lt;/me:ExtraDOSCMethod&gt;
&lt;/molecule&gt;


&lt;molecule id="N2"&gt;
  &lt;atom elementType="N"/&gt;
  &lt;propertyList&gt;
    &lt;property dictRef="me:epsilon"&gt;
      &lt;scalar&gt;48.0&lt;/scalar&gt;
    &lt;/property&gt;
    &lt;property dictRef="me:sigma"&gt;
      &lt;scalar&gt;3.90&lt;/scalar&gt;
    &lt;/property&gt;
    &lt;property dictRef="me:MW"&gt;
      &lt;scalar units="amu"&gt;28.0&lt;/scalar&gt;
    &lt;/property&gt;
  &lt;/propertyList&gt;
&lt;/molecule&gt;
&lt;molecule id="He"&gt;
  &lt;atom elementType="He"/&gt;
  &lt;propertyList&gt;
    &lt;property dictRef="me:epsilon"&gt;
      &lt;scalar&gt;10.22&lt;/scalar&gt;
    &lt;/property&gt;
    &lt;property dictRef="me:sigma"&gt;
      &lt;scalar&gt;2.511&lt;/scalar&gt;
    &lt;/property&gt;
    &lt;property dictRef="me:MW"&gt;
      &lt;scalar&gt;4.04&lt;/scalar&gt;
    &lt;/property&gt;
  &lt;/propertyList&gt;
&lt;/molecule&gt;
&lt;molecule id="Ar"&gt;
  &lt;atom elementType="Ar"/&gt;
  &lt;propertyList&gt;
    &lt;property dictRef="me:epsilon"&gt;
      &lt;scalar&gt;114&lt;/scalar&gt;
    &lt;/property&gt;
    &lt;property dictRef="me:sigma"&gt;
      &lt;scalar&gt;3.47&lt;/scalar&gt;
    &lt;/property&gt;
```

```
      <property dictRef="me:MW">
        <scalar>39.948</scalar>
      </property>
     </propertyList>
   </molecule>

 </moleculeList>


  <reactionList>
              <reaction id="R1">
     <reactant>
       <molecule ref="OH-2" role="modelled" />
     </reactant>
     <reactant>
       <molecule ref="HO2-2" role="excessReactant" />
     </reactant>
     <product>
       <molecule ref="PRC-2" role="modelled" />
     </product>
     <me:MCRCMethod xsi:type="me:MesmerILT">
<me:preExponential units="cm3molecule-1s-1">3.18E-10</me:preExponential>
        <me:activationEnergy units="kJ/mol">0.0</me:activationEnergy>
       <me:TInfinity>298.0</me:TInfinity>
       <me:nInfinity>-0.5</me:nInfinity>

     </me:MCRCMethod>
     <me:excessReactantConc>1e13</me:excessReactantConc>
   </reaction>
              <reaction id="R2">
                   <reactant>
       <molecule ref="PRC-2" role="modelled" />
     </reactant>
     <product>
       <molecule ref="water-2" role="sink" />
     </product>
               <product>
       <molecule ref="O2-t-2" role="sink" />
     </product>
     <me:transitionState>
       <molecule ref="TS-abst-1-t-2" role="transitionState" />
     </me:transitionState>
                <me:tunneling>Eckart</me:tunneling>
     <me:MCRCMethod name="SimpleRRKM"/>
   </reaction>

  </reactionList>

   <me:conditions>
    <me:InitialPopulation>
        <me:molecule ref="OH-1" me:population="1.000" />
        </me:InitialPopulation>
   <me:bathGas>N2</me:bathGas>
   <me:PTs>

<me:PTpair me:units="Torr" me:P="1850" me:T="300" me:precision= "dd" />


   </me:PTs>
  </me:conditions>

  <me:modelParameters>
   <!--Specify grain size directly...-->
```

```
   <me:grainSize units="cm-1">50</me:grainSize>
   <!-- <me:energyAboveTheTopHill>25</me:energyAboveTheTopHill> -->
   <me:automaticallySetMaxEne>5.0e-15</me:automaticallySetMaxEne>
  </me:modelParameters>

  <me:control>
   <me:testDOS />
    <me:printSpeciesProfile />
    <me:testMicroRates />
    <me:testRateConstant />
    <me:printGrainDOS />
    <me:printCellDOS />
    <me:printReactionOperatorColumnSums />
    <me:printTunnellingCoefficients />
    <me:printGrainkfE />
    <me:printGrainBoltzmann />
     <me:printGrainkbE />
     <me:eigenvalues>5</me:eigenvalues>
  </me:control>
</me:mesmer>
```


References

1. Stone, D.; Blitz, M.; Ingham, T.; Onel, L.; Medeiros, D. J.; Seakins, P. W., An instrument to measure fast gas phase radical kinetics at high temperatures and pressures. *Rev. Sci. Instrum.* **2016,** *87* (5).
2. Speak, T. H.; Blitz, M. A.; Stone, D.; Seakins, P. W., A new instrument for time-resolved measurement of HO_2 radicals. *Atmospheric Measurement Techniques* **2020,** *13* (2), 839-852.
3. Onel, L.; Blitz, M. A.; Seakins, P. W., Direct Determination of the Rate Coefficient for the Reaction of OH Radicals with Monoethanol Amine (MEA) from 296 to 510 K. *Journal of Physical Chemistry Letters* **2012,** *3* (7), 853-856.
4. Glowacki, D. R.; Lockhart, J.; Blitz, M. A.; Klippenstein, S. J.; Pilling, M. J.; Robertson, S. H.; Seakins, P. W., Interception of excited vibrational quantum states by O_2 in atmospheric association reactions. *Science (Washington, D. C., 1883-)* **2012,** *337*, 1066-7.
5. Ianni, J. C., A Comparison of the Bader-Deuflhard and the Cash-Karp Runge-Kutta Integrators for the GRI-MECH 3.0 Model Based on the Chemical Kinetics Code Kintecus. In *Computational Fluid and Solid Mechanics*, Bathe, K. J., Ed. Elsevier: Oxford, 2003; pp 1368-1372.
6. Frisch, M. J.; Trucks, G. W.; Schlegel, H. B.; Scuseria, G. E.; Robb, M. A.; Cheeseman, J. R.; Scalmani, G.; Barone, V.; Petersson, G. A.; Nakatsuji, H.; Li, X.; Caricato, M.; Marenich, A.; Bloino, J.; Janesko, B. G.; Gomperts, R.; Mennucci, B.; Hratchian, H. P.; Ortiz, J. V.; Izmaylov, A. F.; Sonnenberg, J. L.; Williams-Young, D.; Ding, F.; Lipparini, F.; Egidi, F.; Goings, G.; Peng, B.; Petrone, A.; Henderson, T.; Ranasinghe, D.; Zakrzewski, V. G.; Gao, J.; Rega, N.; Zheng, G.; Liang, W.; Hada, M.; Ehara, M.; Toyota, K.; Fukuda, R.; Hasegawa, J.; Ishida, M.; Nakajima, T.; Honda, Y.; Kitao, O.; Nakai, H.; Vreven, T.; Throssell, K.; Montgomery, J. A. J.; Peralta, J. E.; Ogliaro, F.; Bearpark, M.; Heyd, J. J.; Brothers, E.; Kudin, K. N.; Staroverov, V. N.; Keith, T.; Kobayashi, R.; Normand, J.; Raghavachari, K.; Rendell, A.; Burant, J. C.; Iyengar, S. S.; Tomasi, J.; Cossi, M.; Millam, J. M.; Klene, M.; Adamo, C.; Cammi, R.; Ochterski, J. W.; Martin, R. L.; Morokuma, K.; Farkas, O.; Foresman, J. B.; Fox, D. B. *Gaussian 09, Revision A.02*, Gaussian: Wallingford CT, 2009.
7. Kanno, N.; Tonokura, K.; Koshi, M., Equilibrium constant of the HO_2-H_2O complex formation and kinetics of HO_2+HO_2-H_2O: Implications for tropospheric chemistry. *Journal of Geophysical Research-Atmospheres* **2006,** *111* (D20), 7.

8. Glowacki, D. R.; Liang, C. H.; Morley, C.; Pilling, M. J.; Robertson, S. H., MESMER: An open-source master equation solver for multi-energy well reactions. *Journal of Physical Chemistry A* **2012,** *116* (38), 9545-9560.
9. Cox, R. A.; Burrows, J. P.; Wallington, T. J., Rate coefficient for the reaction OH + HO_2 = H_2O + O_2 at 1 atmosphere pressure and 308 K. *Chemical Physics Letters* **1981,** *84* (2), 217-221.
10. DeMore, W. B., Rate constant and possible pressure dependence of the reaction OH + HO_2. *Journal of Physical Chemistry* **1982,** *86* (1), 121-126.
11. Braun, M.; Hofzumahaus, A.; Stuhl, F., VUV Flash Photolysis Study of the Reaction of HO with HO_2 at 1 atm and 298 K. *Berichte der Bunsengesellschaft für Physikalische Chemie* **1982,** *86* (7), 597-602.
12. Dransfeld, P.; Wagner, H. G., Comparative Study of the Reactions of ^{16}OH and ^{18}OH with $H^{16}O_2$. *Zeitschrift für Naturforschung A* **1987,** *42* (5), 471-476.
13. Hickson, K. M.; Keyser, L. F., A kinetic and product study of the Cl+HO_2 reaction. *Journal of Physical Chemistry A* **2005,** *109* (31), 6887-6900.
14. Riffault, V.; Bedjanian, Y.; Le Bras, G., Kinetics and mechanism of the reaction of Cl atoms with HO_2 radicals. *Int. J. Chem. Kinet.* **2001,** *33* (5), 317-327.
15. Dobis, O.; Benson, S. W., Reaction of the ethyl radical with oxygen at millitorr pressures at 243-368 K and a study of the Cl+HO_2, ethyl+HO_2, and HO_2+HO_2 reactions. *J. Am. Chem. Soc.* **1993,** *115* (19), 8798-8809.
16. Lee, Y. P.; Howard, C. J., Temperature-dependence of the rate-constant and the branching ratio for the reaction Cl+HO_2. *J. Chem. Phys.* **1982,** *77* (2), 756-763.
17. Cattell, F. C.; Cox, R. A., Pressure-dependence of the reactions of HO_2 with Cl and ClO. *Journal of the Chemical Society-Faraday Transactions II* **1986,** *82*, 1413-1426.
